# An intensity ratio of interlocking loops determines circadian period length

**DOI:** 10.1093/nar/gku701

**Published:** 2014-08-13

**Authors:** Jie Yan, Guangsen Shi, Zhihui Zhang, Xi Wu, Zhiwei Liu, Lijuan Xing, Zhipeng Qu, Zhen Dong, Ling Yang, Ying Xu

**Affiliations:** 1 Center for Systems Biology, Soochow University, Suzhou 215006, China; 2 MOE Key Laboratory of Model Animal for Disease Study, Model Animal Research Center, Medical School of Nanjing University, Nanjing 210061, China; 3 School of Mathematical Sciences, Soochow University, Suzhou 215006, China; 4 Collaborative Innovation Center for Genetics and Development, Fudan University, Shanghai 200433, China

## Abstract

Circadian clocks allow organisms to orchestrate the daily rhythms in physiology and behaviors, and disruption of circadian rhythmicity can profoundly affect fitness. The mammalian circadian oscillator consists of a negative primary feedback loop and is associated with some ‘auxiliary’ loops. This raises the questions of how these interlocking loops coordinate to regulate the period and maintain its robustness. Here, we focused on the REV-ERBα/*Cry1* auxiliary loop, consisting of Rev-Erbα/ROR-binding elements (RORE) mediated *Cry1* transcription, coordinates with the negative primary feedback loop to modulate the mammalian circadian period. The silicon simulation revealed an unexpected rule: the intensity ratio of the primary loop to the auxiliary loop is inversely related to the period length, even when post-translational feedback is fixed. Then we measured the mRNA levels from two loops in 10-mutant mice and observed the similar monotonic relationship. Additionally, our simulation and the experimental results in human osteosarcoma cells suggest that a coupling effect between the numerator and denominator of this intensity ratio ensures the robustness of circadian period and, therefore, provides an efficient means of correcting circadian disorders. This ratio rule highlights the contribution of the transcriptional architecture to the period dynamics and might be helpful in the construction of synthetic oscillators.

## INTRODUCTION

Circadian clocks are self-sustained oscillators that regulate the temporal organization of physiology, metabolism and behavior to adapt to the 24-h solar day. In many organisms, mutants conferring altered circadian period are associated with physiological disorders and decreased lifespan (1–3). The central mechanism of the mammalian circadian clock is a negative primary feedback loop that involves the transcriptional activator genes *Clock* and *Bmal1*, and three *Period* (*Per1*–*Per3*) and two *Cryptochrome* (*Cry1* and *Cry2*) repressor genes (4). CLOCK and BMAL1 are basic helix-loop-helix periodic acid-Schiff-domain-containing transcription factors that activate *Per* and *Cry* gene transcription (5–9). The resulting PER and CRY proteins accumulate in the cytosol, and following various modifications, are then translocated into the nucleus in which they inhibit CLOCK/BMAL1-mediated transcription, thereby establishing a negative feedback loop (10). Thus, the delayed nuclear accumulation of PERs and CRYs is thought to be the primary factor that determines the period length.

Various auxiliary loops (ALs) also participate in the regulation of mammalian circadian clocks. Previous studies validated the existence of the REV-ERB/ROR-binding element (RORE) in the first intron of *Cry1* (11,12). Thus, *Cry1* expression is positively autoregulated via the inhibition of its repressor *Rev-erbα*, which forms a positive AL (RORE loop). Some nuclear receptors, exhibiting circadian-like patterns of expression (13), may contribute to the circadian clock via other ALs. These ALs provide multiple entry points to modulate the circadian clock to adapt to environmental variations which may affect the oscillation period. However, when deprived of a rhythmic input, the free-running period can usually maintain the period close to 24 h over a wide range of situations, including environmental diversity and gene mutations. Therefore, two important questions arise: (i) how do these ALs coordinate with the primary loop to modulate the period length? (ii) How does the system with more than one loop maintain the robustness of the free-running period?

Mathematical modeling is a powerful tool to both interpret the circadian networks of various organisms (14–19) and to obtain a general understanding of how biological oscillators function (20,21). Here, to address the above questions, we used a combined mathematical-experimental approach to explore how the positive RORE loop, which is a typical AL (RORE loop) in circadian systems, coordinates with the primary loop to modulate the period through transcriptional regulation. Previous models addressing the role of RORE loop have substantially contributed to several aspects of the circadian clock. Nevertheless, the models developed here differ from other models in two major respects. First, we used a currently identified positive loop through the *Cry1*, instead of widely used negative AL through *Bmal1*, to study the mechanism of period determination. The negative AL through *Bmal1* is usually thought to stabilize the oscillation of the circadian clock (16,22). Second, according to the recent experimental evidence (11), we used the addition rule to describe the co-regulation of the *cis*-elements. This addition rule can provide the accurate description of the amplitude contribution, which is not available under the traditional multiply rule (17,22–24). The above differences enabled our model to illustrate the role of amplitudes in period dynamics and achieve reasonably qualitative predictions.

## MATERIALS AND METHODS

### Mathematical models

Supplementary information provides detailed information about conceptual models, comprehensive models, the numerical simulations and theoretical analysis.

### Generation of single- and double-mutant mice

*Clock^Δ19^* (25) were originally obtained from the Takahashi laboratory at Northwestern University. *Rev-erbα* knockout mice (26) were obtained from Ueli Schibler lab in University of Geneva. *Per2* knockout mice (27) were obtained from the Jackson laboratory. All other mutant mice were described in our previous works (28–30).

### Animal care and behavioral analysis

All of the animals were backcrossed at least six generations prior to the first pilot study to assure their C57BL/6J backgrounds. Measurements of the free running period were performed as previously described in (32). Mice were placed in individual cages that were equipped with running wheels in light-tight ventilation chambers with timer-controlled lighting. Wheel running activities were monitored over a 12:12-h LD cycle for 7 days and followed in constant darkness (DD) for up to 4 weeks. Analysis of circadian parameters was performed using ClockLab, and the period was calculated from 8 days to 21 days after constant darkness (Actimetrics Software).

All animal studies were carried out in an Association for Assessment and Accreditation of Laboratory Animal Care (AAALAC International)-accredited Specific Pathogen Free animal facility, and all animal protocols were approved by the Animal Care and Use Committee of the Model Animal Research Center, the host for the National Resource Center for Mutant Mice in China, Nanjing University.

### Tissue collection, RNA isolation, mRNA quantitative polymerase chain reaction analyses

Mice of the indicated genotypes were entrained to a 12–12-h light-dark cycle for at least 7 days before being transferred to DD. Tissues were collected at 4-h intervals on the first day of DD at circadian times (CTs) 0, 4, 8, 12, 16, 20 and 24, where CT12 corresponds with the onset of a subjective night. Each time point has an average three to four mice for each genotype. RNA isolation and quantitative reverse transcriptase-polymerase chain reaction (qRT-PCR) (including primers for mRNA profiling) were carried out essentially as previously described (30).

### The amplitude of *Per1* mRNA and *Bmal1* mRNA

The amplitude of *Per1* mRNA is the difference between the peak and the trough of the *Per1* expression profile. To compare the amplitudes of *Per1* mRNA in different genotypes, the amplitude of *Per1* mRNA in WT was normalized to 1, and the expression levels of other genotypes were plotted as percentages of WT. This also was done for the amplitude of *Bmal1*. The formula was written as Amplitude = {mutant peak/wild type (peak – trough)} – {mutant trough/wild type (peak – trough)}.

### Transfection and luminescence recording

U2OS-Luciferase reporter cells were cultured in Dulbecco's modified Eagle's medium containing 10% serum and penicillin–streptomycin in 35 mm plates. Expression plasmids for *Rev-erbα*, *Cry1* and *Per2* genes were described previously (29). Lipofectamine 2000 (Invitrogen) and Genescort (Wisegen) were used for transfection, according to the manufacturer's instructions. 60 hours post-transfection, we replaced this media with an HEPES-buffered medium supplemented with luciferin (1 μM) (Promega) and B-27 supplements (Invitrogen), and the plates were sealed with an optically clear film (Applied Biosystems). Luminescence was recorded by LumiCycle.

We then used the LumiCycle circadian data analysis program (Actimetrics) to analyze the bioluminescence data. The data were first detrended by subtracting a baseline (24-h running average), and, subsequently, fit to a sine wave to obtain the period length of the oscillation.

## RESULTS

### Constructing a conceptual model of period dynamics

**Figure 1.**
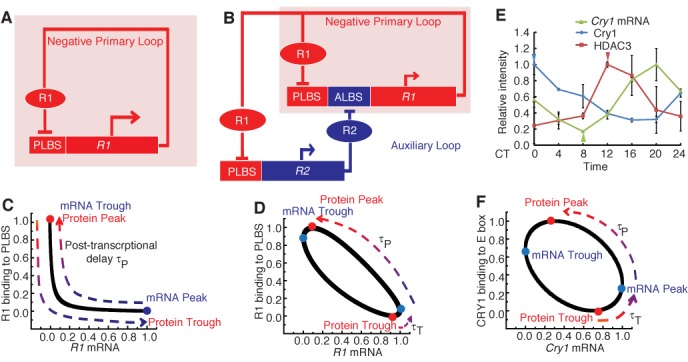
The auxiliary loop coordinates with the primary loop to provide a transcriptional contribution. (**A**) Schematic representation of the negative primary feedback loop. (**B**) Schematic representation of the cooperation between the negative primary and positive auxiliary loops (AL: auxiliary loop; ALBS: auxiliary loop binding sites; PLBS: primary loop binding sites). (**C**) Phase plot between *R1* mRNA expression and R1 binding to the PLBS. The R1 protein peak coincides with the *R1* mRNA trough and R1 protein trough coincides with the *R1* mRNA peak. (**D**) Phase plot between *R1* mRNA expression and R1 binding to the PLBS after introduction of the auxiliary loop. The *R1* mRNA peak is separated from the R1 protein trough by the transcriptional delay, τ_T_. (**E**) The expression of the *Cry1* mRNA (green) and the enrichment of CRY1 binding to E-box regions of the *Cry1* (blue) and HDAC3 binding to RORE region of *Cry1* (red) in mouse liver tissue. The result showed that the trough of *Cry1* mRNA (the green triangle) is between the peak of CRY1 protein (the blue triangle) and the peak of HDAC3 (the red triangle) protein. Expression level of *Cry1* was measured by qRT-PCR and normalized to *Gapdh*. Relative RNA levels were expressed as a percentage of the maximal value for each experiment. Each value represents the mean ± SD (*n* = 3). Chromatin samples from mouse liver tissue were analyzed by ChIP (each time point represents an average of three to four mice). **(F)** Phase plot between *Cry1* mRNA expression and CRY1 protein binding to the E-box, both of which are fitted by a sine function.

To gain a system-level understanding of transcriptional period regulation, we first refined the core circadian network as a schematic model (Figure 1A and B) rather than specifying individual genes. By comparing conditions with or without a positive AL, the conceptual model has indicated that an AL can markedly contribute to the mammalian circadian period (see Sections 1.1 and 1.2 of the Supplementary Information, and Supplementary Table S1). We were then able to extend the results to a comprehensive model with multiple genes. To focus on transcriptional influence, we simplified the interval between mRNA expression and the corresponding protein binding to *cis*-elements (post-transcriptional processes) as a fixed time delay, τ_P_.

Figure 1A schematizes the system with only the negative primary feedback loop. In this situation, the expression of the repressor gene *R1* is enhanced by primary loop binding sites (PLBS). The resulting R1 protein is translocated into the nucleus and binds to its own PLBS to repress PLBS-mediated transcription. As previously assumed, the duration from the peak of *R1* mRNA to the peak of R1 protein binding to the PLBS is approximately equal to τ_P_ hours (Figure 1C). Because the *R1* gene is solely inhibited by the R1 protein, when R1 binding to the PLBS reaches its trough, the transcription activity peaks. Thus, the R1 protein trough is at the peak of *R1* mRNA. Therefore, the duration from R1 protein trough to R1 protein peak is approximately τ_P_ hours.

When an AL (for example, the positive RORE AL) is introduced into the conceptual model, the transcription of the repressor gene *R1* (for example, *Cry1*) is dually regulated by the PLBS and an AL binding site (ALBS), such as the RORE from the AL (Figure 1B). The AL (RORE loop) participates in *R1* (*Cry1*) transcription through ALBS (RORE); protein R1 (CRY1) represses the PLBS-mediated transcription of *R2* (*Rev-erbα*), and R2 (REV-ERBα) feeds back to inhibit *R1* (*Cry1*) by binding to the ALBS (RORE). This positive feedback loop results in a distinct feature of the system: it separates the *R1* mRNA phase from the PLBS activity peak. It is worthwhile to note that the primary loop in our model is a simplification of the E-box-dominated negative feedback loops. PLBS in our model is basically the E-box, but with the phase delay due to other *cis*-elements (for example, D-box). According to previous studies, the phase of RORE is later than that of the combination of canonical E-box and D-box (11). If we assume that the peak activity of the ALBS occurs later than that of the PLBS, then a delayed *R1* mRNA peak is ensured (like *Cry1* mRNA). As shown in Figure 1D, unlike the primary loop-only scenario, the duration from the R1 protein trough to the R1 protein peak is separated into two parts upon the inclusion of a positive AL: a transcriptional delay from the R1 protein trough to the *R1* mRNA peak and a post-transcriptional delay from the *R1* mRNA peak to the R1 protein peak. Therefore, the AL (RORE) confers a time delay on the primary loop by providing a transcriptional delay, τ_T_, to modulate circadian cycle length. A similar result was obtained within the comprehensive model which includes multiple repressors (Supplementary Figure S1A and B).

We experimentally explored the conceptual model to visualize the contribution of the AL to the period length. We measured the profiles of CRY1 binding to the E-box of the *Cry1* gene and histone deacetylase 3 (HDAC3) binding to the RORE of *Cry1* using chromatin immunoprecipitation (ChIP). HDAC3 has been shown to recruit (REV-ERBα)-containing complexes to the RORE and repress the transcription of *Cry1* expression (31). *Cry1* mRNA was measured using qPCR in mouse liver tissues to characterize the features of the separation of the repressor binding trough from the peak of *R1* mRNA expression in the mammalian circadian clock. The results indicated that the *Cry1* mRNA peak falls between the troughs of CRY1 and HDAC3 (Figure 1E). CRY1 and HDAC3, respectively, inhibit the activity of the E-box and RORE. Therefore, the troughs of CRY1 and HDAC3 correspond to the peaks of E-box activity and RORE activity. Since the peak of *Cry1* mRNA is between the peaks of E-box and RORE, it is reasonable to speculate that this delay in *Cry1* expression reflects the participation of circadian RORE behavior in the expression of *Cry1*. Therefore, the RORE-mediated transcriptional loop contributes to the period length by regulating the time duration between the trough of CRY1 protein binding to the E-box and peak of *Cry1* mRNA expression (which is symmetric to the peak of CRY1 protein binding to the E-box and the trough of *Cry1* mRNA expression) (Figure 1F).

The theoretical analysis and experimental results support the hypothesis that the AL facilitates a phase change in repressor expression in the primary loop through which a time delay of its self-inhibition can be regulated. This action then contributes to the period.

### The intensity ratio between the primary loop and the AL modulates the cycle length

To explain how the interplay between the primary and ALs influences the circadian period, we used the conceptual model shown in Figure 1B to demonstrate the kinetics of circadian rhythms. Although this model is simple, it retains the basic architecture of multiple loops and gives rise to a novel property of period dynamics: the intensity ratio between the primary and ALs is strongly associated with the transcriptional delay, τ_T_, and, in turn, contributes to the period length.

**Figure 2.**
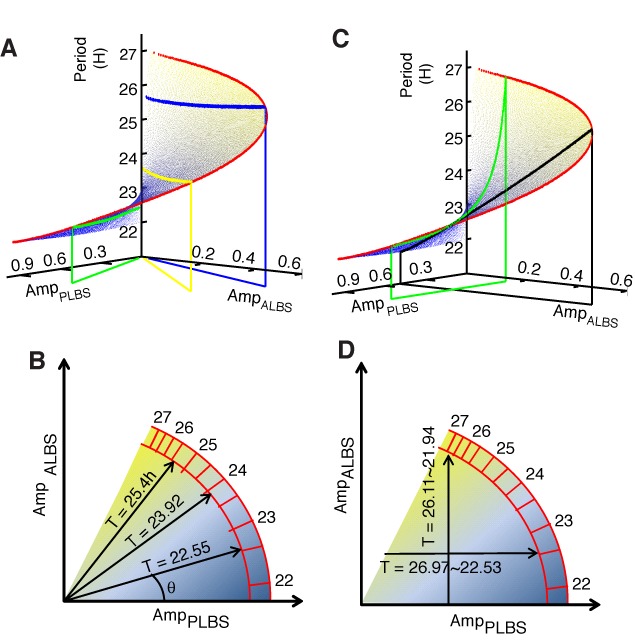
The intensity ratio between feedback loops regulates the period length. (**A**) This surface exhibits a spiral staircase-like shape in which the height (period length) increases as the rotation angle increases. The *x*-axis and the *y*-axis represent the amplitudes of PLBS activity and ALBS activity, respectively. Three vertical planes, which represent Amp_PLBS_ / Amp_ALBS_ = 4.2888 (green), Amp_PLBS_ / Amp_ALBS_ = 1.3764 (yellow) and Amp_PLBS_ / Amp_ALBS_ = 0.6682 (blue), intersect with the surface in three horizontal lines at periods of ∼22.55 h (green), 23.92 h (yellow) and 25.4 h (blue). (**B**) The color bar indicates the period variation in the PLBS–ALBS amplitude plane. The rotation angle θ is determined by the amplitude ratio of PLBS activity to ALBS activity (cot (θ) = Amp_PLBS_ /Amp_ALBS_). The period length increases as θ increases. (**C**) Two vertical planes, which represent PLBS = 0.5 (blue) and ALBS = 0.2 (red), intersect with the period surface in two curves (black curve). These two curves exhibit large height variations (from 21.94 h to 26.11 h when PLBS = 0.5 and from 22.53 h to 26.97 h when ALBS = 0.2). (**D**) The vertical arrow crosses over several color zones (from 21.94 h to 26.11 h), which indicate that a fixed PLBS cannot determine the period length. Also, the horizontal arrow crosses over several color zones (from 22.53 h to 26.97 h), which indicate that a fixed ALBS cannot determine the period length.

We first used a theoretical analysis to deduce the length of transcriptional delay. It turns out to be approximately determined by the amplitude ratio of PLBS activity (denoted as Amp_PLBS_, i.e. the intensity of the primary loop) to ALBS (RORE) activity (denoted as Amp_ALBS_, i.e. the intensity of the AL), as follows: τ_T_ = *f* (Amp_PLBS_/Amp_ALBS_), where *f* is a decreasing function (see Section 1.3 of the Supplementary Information). To intuitively link the intensities of the primary and ALs to the period length, we manipulated the PLBS and ALBS (RORE) activity in silicon. The relationship between the period and the amplitudes of the binding element activity is illustrated in Figure 2A. The vertical axis indicates the period length (H), and the two horizontal axes represent the oscillation amplitudes of the binding element (PLBS and ALBS) activity. The surface exhibits a spiral staircase-like shape, in which the height of the surface (period length) increases as the rotation angle increases, and any radial line approximates a contour line on the surface (where all points on the line share a common altitude). For example, the green vertical plane, whose slope equals 4.2888, intersects with the surface at the horizontal green line (about 22.55 h). Similarly, the yellow and blue vertical planes, whose slopes are 1.3764 and 0.6682, intersect with the surface at the yellow horizontal line (about 23.92 h) and the blue horizontal line (about 25.4 h), respectively. The schematic diagram in Figure 2B intuitively indicates the relationship between the period length and the amplitude ratio: the rotation angle θ is determined by cot (θ) = Amp_PLBS_/Amp_ALBS_, and the period length increases as the rotation angle increases. Figure 2C and D illustrates that when Amp_PLBS_ = 0.5, T varies from 21.94 h to 26.11 h, whereas when Amp_ALBS_ = 0.2, T varies from 22.53 h to 26.97 h. The following two concepts arise from this model. (i) The circadian period can be approximated by Amp_PLBS_/Amp_ALBS_ under the assumption that the post-transcriptional delay is a constant. (ii) A single-activity amplitude is not sufficient to regulate the period.

To investigate whether the ratio-regulating prediction still holds within other possible biological oscillators, we performed the silicon simulations in a broader range of parameter space. The results show that the ratio law still works in a wide range of period length (Supplementary Figure S2). It implies that the oscillator with this topological structure should have a similar ratio-regulating pattern.

Because the analysis and simulation presented herein are based on a minimalist model, we further extended our analysis under more realistic conditions. We performed computational simulations in a comprehensive model involving *Bmal1*, *Per1*, *Cry1*, *Per2*, *Cry2* and *Rev-erbα* to verify the ratio hypothesis (Supplementary Figure S3; see Sections 2.1 and 2.2 of the Supplementary Information, Supplementary Table S2). Also, our comprehensive model generated autonomous circadian oscillations and reproduced the period length well under different conditions (Supplementary Table S3). The comprehensive model places the expressions of the genes approximately in-phase in WT case (Supplementary Table S4). Therefore, we believe that this validated model can provide a qualitatively predictive tool for studying the properties of the circadian clock.

**Figure 3.**
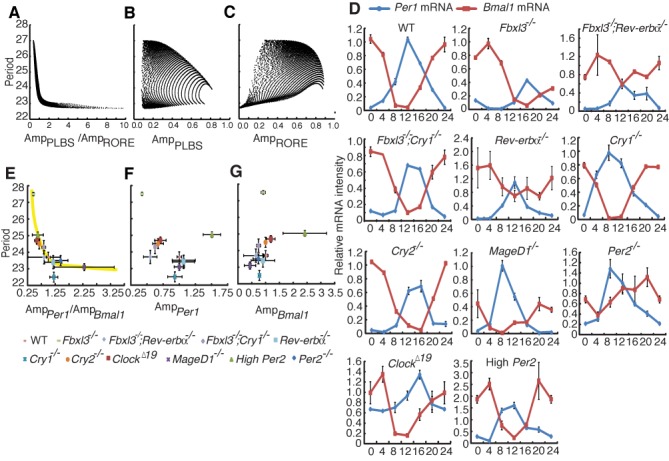
Validation of the ratio hypothesis using a comprehensive model and experimental results. (**A–C**) Computational simulations in a comprehensive mathematical model. The periods are shown as a function of the amplitude ratio of PLBS activity to RORE activity (**A**), the amplitude of PLBS activity (**B**) and the amplitude of RORE activity (**C**). (**D**) The expression profiles of the *Per1* (blue) and *Bmal1* (red) clock genes in liver tissues from wild-type, *Fbxl3^−/−^*, *Fbxl3^−/−^; Rev-erbα^−/−^, Fbxl3^−/−^; Cry1^−/−^, Rev-erbα ^−/−^*, *Cry1^−/−^*, *Cry2^−/−^*, *Maged1^−/−^*, *Per2^−/−^*, *Clock^Δ19^* and high copy *Per2* mice were determined using qRT-PCR and were normalized to *Gapdh*. We calculated the relative amplitude of *Bmal1* or *Per1* between liver tissues from the indicated genotypes and wild-type mice, and the expression levels were plotted as percentages of the WT amplitude in each paired experiment. Each value represents the mean ± SD (*n* = 3). (**E–G**) Summary of the experimental studies. The free running periods are shown as a function of the amplitude ratio of *Per1* mRNA to *Bmal1* mRNA (**E**), the amplitude of *Per1* mRNA (**F**) and the amplitude of *Bmal1* mRNA (**G**). The amplitude of *Per1* mRNA or *Bmal1* mRNA means the difference between the maximal expression level and the minimal expression level. Each genotype is represented by the indicated symbol. The each error bar indicates the standard deviation.

Similar to the conceptual model, the simulation results indicated a clear negative relationship, i.e. the circadian period increases as the amplitude ratio of PLBS activity (denoted as Amp_PLBS_) to RORE activity (denoted as Amp_RORE_) decreases (Figure 3A). However, neither Amp_PLBS_ nor Amp_RORE_ alone exhibits any clear monotonic relationship to the period (Figure 3B and C). These results suggest that the participation of AL provides the possibility to fine-tune the period of an oscillator.

### Negative relationship between Amp*_Per1_*/Amp*_Bmal1_* and the period length

We then analyzed 10 strains of mutant mice to experimentally test the simulated ratio hypothesis. In this study, we focus on how the positive RORE loop affects the period length through the repressor genes in the negative primary feedback loop. According to previous studies, the RORE can delay the expression of *Cry1*, because the phase of RORE is later than that of the combination of canonical E-box and D-box (11,12). Similar to their studies, we used the canonical E-box and D-box as a whole to stand for the negative primary feedback loop (PLBS) in our simulation. Since *Per1* is driven by both the canonical E-box and D-box (32,33), the amplitude of *Per1* mRNA is suited to be the intensity indicator of the negative primary feedback loop. We thus selected *Per1* as a biomarker for the intensity of primary loop. The amplitude of *Bmal1* mRNA which is driven by RORE activity can be used as the indicator to represent the intensity of the positive auxiliary feedback loop (AL). The oscillations of *Per1* and *Bmal1* were approximately antiphase in wild-type liver tissue, and the pattern alterations in each mutant mouse strain were similar to those from previous reports (Figure 3D). The amplitudes (the difference between trough and peak values) of *Per1* (denoted as Amp*_Per1_*) and *Bmal1* (denoted as Amp*_Bmal1_*) were calculated for each mutant mouse strain, and their ratios correlated with the wheel-running period (Figure 3E), which is consistent with the model predictions (Figure 3A). This result demonstrates that there is a clear negative relationship between Amp*_Per1_*/Amp*_Bmal1_* and the period length. Furthermore, neither the amplitude of *Per1* nor *Bmal1* mRNA alone exhibited any clear monotonic relationship to the periods (Figure 3F and G). In some case such as *Maged1* knockout mice, there is a wide range of error bars. One interpretation is that error bars stem from the fact that the amplitude that is the difference between the maximal expression level and minimal expression level has the double relative error, the ratio of the amplitudes further expands the relative error and, in the end, overestimates the strength of unreliability. Even though, these error bars do not disrupt our conclusions. As shown in Figure 3E–G, the points having large error bars are located in the short period area, where the period length does not have notable change in a wide range of amplitude ratios (from 1.5 to 3.75). Also, from the simulation results, the short period is not sensitive to the intensity ratio.

These results confirm that the balance between the primary loop and the AL (i.e. the RORE loop through *Cry1*) determines the period length. The only exception is the ratio observed in *Cry1^−/−^* mice. However, this trend was expected because the removal of *Cry1* destroys the AL of REV-ERBα/*Cry1*. In this study, our prediction is based on the interaction between the negative primary feedback loop and the positive RORE loop (REV-ERBα/*Cry1* loop). In *Cry1^−/−^* mice, this positive RORE loop (REV-ERBα/*Cry1* loop) vanishes due to the knockout of *Cry1* gene. Therefore, the point which represents *Cry1^−/−^* mice is not located right on the decreasing curve. In fact, REV-ERBα/*Cry1* loop may not be the only positive RORE loop, and results from systems biology studies have suggested that *Per1*, *Per2* and *Cry2* might also contain ROREs (34) and be able to form additional positive ALs with REV-ERBα (as the structure of REV-ERBα/*Cry1* loop). The role of the REV-ERBα/*Cry1* loop in the period determination can be compensated by these ALs. Therefore, destroying the REV-ERBα/*Cry1* AL will result in only partial deviation from, rather than complete disruption of the ratio rule.

### Manipulating the circadian period in a predictable fashion

**Figure 4.**
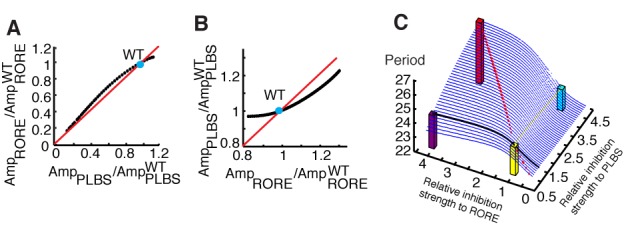
Period robustness through a coupling effect. (**A**) The amplitude of PLBS activity (*x*-axis) is transmitted to the amplitude of RORE activity (*y*-axis) as the strength of inhibition to the PLBS is manipulated. The blue point represents the WT case, and the red line indicates 1:1 complete transmission. Amp_PLBS_ and Amp_RORE_ represent the resultant amplitudes of PLBS and RORE activity. Amp_PLBS_^WT^ and Amp_RORE_^WT^ represent the amplitudes of PLBS and RORE activity in the WT case. (**B**) The amplitude of RORE activity (*x*-axis) is partially transmitted to the amplitude of PLBS activity (*y*-axis) as the strength of inhibition to the RORE is manipulated. (**C**) The relationship between the period length (*z*-axis) and the strengths of inhibition to the PLBS (*x*-axis) and RORE (*y*-axis). If the mutation only alters the strength of inhibition in one loop (the yellow line indicates the negative primary loop, and the black line indicates the RORE auxiliary loop), the period is not greatly altered (the cyan bar and the purple bar). If the mutation alters both loops, the period can be significantly altered (the red dashed line on the ridge, the red bar). The yellow bar indicates the WT.

In the above cases, we did observe variations of the period due to the changes in the amplitude ratio. However, the regulation of the ratio between the PLBS and RORE can aid in ensuring period robustness because of a coupling effect between loops. In the integrated loops, the activity of the PLBS and RORE are partially associated with each other. This association indicates that the amplitude variation of one binding element results in a responding change in the other elements. If a change occurs in the strength of PLBS inhibition, the resultant amplitude variation for the PLBS can be completely transmitted to the RORE amplitude (Figure 4A); correspondingly, the change in the amplitude of the RORE, which results from the variation of the inhibition to the RORE, can be partially transmitted to the PLBS amplitude (Figure 4B). Therefore, the ratio modulation is a robust feature, implying that the oscillation period cannot be dramatically altered through one binding element alone. Subsequently, we observed the association between the period and the strengths of inhibition to binding element activity (Figure 4C). In the figure, the vertical axis represents the period length, and the two horizontal axes represent the individual strengths of the inhibition to the PLBS and RORE. When change is applied to the inhibition to the PLBS, the period of oscillation remains approximately constant (the yellow line). Similarly, the period is slightly altered (the black line) if variation of the inhibition only occurs for the RORE. Thus, this intensity–balance modulation provides the period robustness via loop coupling effect.

The simulation results also indicate that if the strengths of inhibition to both the PLBS and RORE increase, the period will significantly increase along a ridge (the red dashed line) (Figure 4C).

**Figure 5.**
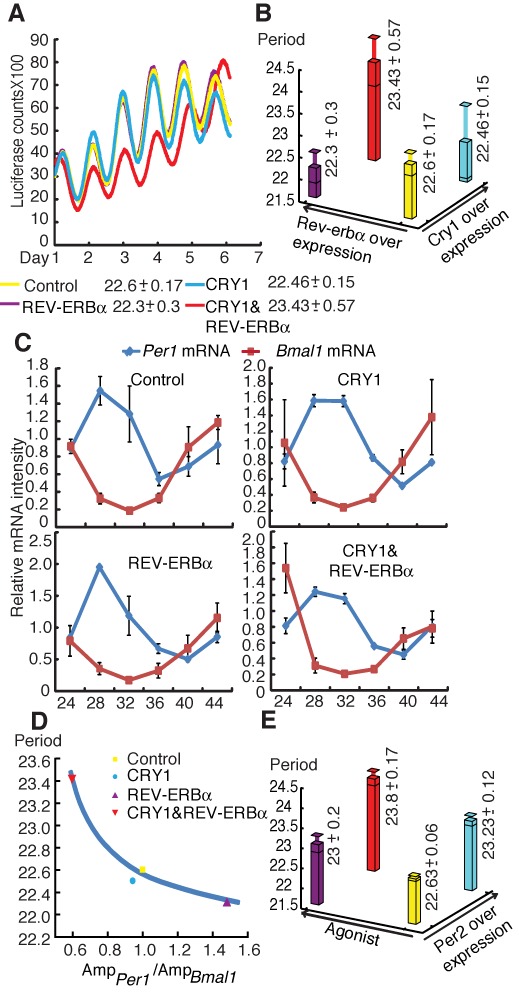
Manipulating the circadian period through the disruption of the transmitting effect. (**A**) Bioluminescence results from a representative experiment 36 h after cells were cotransfected with the indicated expression vectors. All experiments were repeated at least three times, and at least three replicates were performed for each group. (**B**) The relationship between period length (*z*-axis), and REV-ERBα overexpression (*x*-axis) and CRY1 overexpression (*y*-axis). The yellow bar indicates the period of the control group with blank vectors (22.6 h). Upon CRY1 or REV-ERBα overexpression, the period remains at 22.46 h (the cyan bar) or 22.3 h (the purple bar). Upon the overexpression of both CRY1 and REV-ERBα, the period increases to 23.46 h (the red bar). (**C**) Expression profiles of the *Bmal1* (red) and *Per1* (blue) clock genes in U2OS cells with the indicated vectors were measured by qPCR and normalized to *Gapdh*. The expression levels were plotted as percentages of the control amplitude in each paired experiment. Each value represents the mean ± SD (*n* = 3). (**D**) The periods of cells transfected with the indicated vectors are shown versus the amplitude ratio of *Per1* mRNA to *Bmal1* mRNA. Each period represents the mean of three experiments. (**E**) The relationship between period length (*z*-axis), and PPARα agonist overexpression (*x*-axis) and PER2 overexpression (*y*-axis). The period of the control group is 22.63 h, which is indicated by the yellow bar. Upon PER2 overexpression or agonist added only, the period remains at 23 h (cyan bar) or 23.23 h (the purple bar). Upon the overexpression of both PER2 and agonist, the period increases to 23.8 h (the red bar).

To confirm that targeting both the PLBS and RORE can modulate the period, we used U2OS (human osteosarcomas) circadian reporter cells that had previously been successfully employed in various circadian period assays (35). We were particularly interested in understanding the combined effect of CRY1 and REV-ERBα overexpression because we anticipated that this effect might alter the intensity ratio of the primary loop to the AL, thereby leading to period elongation. Notably, overexpression of CRY1 (1.5 μg) or REV-ERBα (1.5 μg) alone did not affect the period; only the expression of CRY1 and REV-ERBα in combination elongated the period length (Figure 5A and G), as measured using LumiCycle assays. We further examined the profiles of *Per1* and *Bmal1* mRNA in each evaluated group over the course of the day because LumiCycle data did not well reflect a real amplitude value (Figure 5C). The *Per1* and *Bmal1* amplitude ratios were negatively related to the period length (Figure 5D), suggesting that the ratio controls the period length, as qualitatively predicted by the model and discussed above.

To extend this hypothesis to the other potential AL, we examined the combined effect of PER2 and PPARα on U2OS cell lines. *Pparα* transcription is induced by CLOCK and BMAL1 via an intronic E-box-rich region (36), and this rhythmic nuclear receptor (13) regulates lipid metabolism and energy homeostasis (37). *Per2* promoter contains the PPARα-binding element PPRE, which activates *Per2* transcription (38), implying the existence of an AL between *Per2* and *PPARα*. We, therefore, predicted a similar scenario as observed for CRY1 and REV-ERBα, although an AL between PER2 and PPARα has not yet been confirmed. We found that a combination of PER2 and PPARα agonists enhanced the variation of the period (Figure 5E and Supplementary Figure S4), suggesting that targeting multiple loops may aid in altering the period length, as indicated by the model prediction.

**Figure 6.**
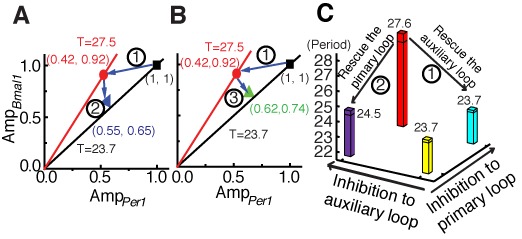
The circadian period can be restored by adjusting the amplitude ratio via transmitting-effect. (**A**) The blue arrows represent the direction of destruction from WT (black square) to *Fbxl3^−/−^* (red circle) mice and the direction of rescue from *Fbxl3*^−/−^ to *Fbxl3^−/−^; Rev-erbα^−/−^* (blue triangle) mice within the *Per1* mRNA-*Bmal1* mRNA amplitude plane. (**B**) The blue arrows represent the direction of destruction from WT (black square) to *Fbxl3^−/−^* (red circle) mice and the direction of rescue from *Fbxl3*^−/−^ to *Fbxl3^−/−^; Cry1^−/−^* (green triangle) mice within the *Per1* mRNA-*Bmal1* mRNA amplitude plane. (**C**) The circadian period can be restored by rescuing only one loop. The yellow bar indicates the period of the WT mice. Arrow 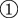
represents the rescue way from *Fbxl3*^−/−^ (the red bar) to *Fbxl3^−/−^; Rev-erbα^−/-^* (the cyan bar), by releasing the compulsions in the auxiliary loop. Arrow 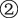
represents the rescue way from *Fbxl3*^−/−^ (the red bar) to *Fbxl3^−/−^; Cry1^−/−^* (the purple bar), by releasing the compulsions in the negative primary feedback loop.

This ratio-regulating pattern can also provide flexibility for circadian period recovery and suggests that the circadian period can be restored through adjustments in the amplitude ratio. *Fbxl3* knockout mice have an extremely long period (29), and it can be restored by knocking out *Rev-erbα* or *Cry1*. To observe the period recovery routes from *Fbxl3^−/−^* (27.5 h) to *Fbxl3^−/−^*; *Rev-erbα^−/−^* (23.7 h) and *Fbxl3^−/−^*; *Cry1^−/−^* (24.5 h) mice, we plotted their amplitudes in the *Per1* mRNA–*Bmal1* mRNA amplitude plane (Figure 6A and B). Points lying on a given radical line in the amplitude plane exhibit an identical ratio. According to the aforementioned ratio hypothesis, the oscillators that correspond to these points exhibit a similar period. For example, phenotypes with amplitudes located near the black radical line will exhibit periods similar to that of WT mice. Theoretically, restoring the amplitudes of a destroyed clock to any point on the black radical line should be sufficient to rescue the period. Experimentally, the amplitudes observed in both *Fbxl3^−/−^*; *Rev-erbα^−/−^* (23.7 h) and *Fbxl3^−/−^*; *Cry1^−/−^* (24.5 h) mice were close to the black radical line, and their periods were also similar to that of WT mice. Moreover, note that the recovery directions (arrow 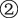 in Figure 6A and arrow 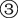 in Figure 6B) of the amplitude ratios in the rescue cases differed from the destruction direction (arrow 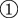 in Figure 6A and B) observed in *Fbxl3^−/−^* mice. This pattern provides flexibility for circadian period recovery and suggests that the circadian period can be restored through adjustments in the amplitude ratio rather than through the wholesale recovery in all destruct variations.

Our previous studies indicated that the negative primary feedback loop and the auxiliary feedback loop were known to be simultaneously targeted in *Fbxl3* knockout mice (29). The improper period is due to the destruct occurred in both loops. Releasing the destruct in the negative primary feedback loop (the deletion of *Cry1* in an *Fbxl3*-null background) or the auxiliary feedback loop (the deletion of *Rev-erbα*) can rescue the amplitude ratio through the coupling effect followed by the recovery of the period. Arrow 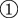 in Figure 6C illustrates the recovery of the period through rescuing the auxiliary loop (*Fbxl3^−/−^*; *Rev-erbα^−/−^* mice, 23.7 h). Similarly, releasing the primary loop (*Fbxl3^−/−^*; *Cry1^−/−^* mice, 24.5 h) can also partially rehabilitate the period length (Arrow 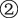 in Figure 6C). Furthermore, if the improper period is caused by unclear reasons, targeting both the primary and ALs may aid in destructing the amplitude ratio and thus rescuing the period. Therefore, rescuing or destructing the intensity ratios of the primary loop to the AL becomes an important issue in designing strategies to correct circadian rhythmic disorders.

## DISCUSSION

In this study, we mainly focus on the mechanism of period determination and find an unexpected and non-intuitive rule that the intensity ratio of the negative primary loop to the positive AL can regulate the period length. To investigate whether this loop-coupling topological structure determines the ratio-regulating pattern, we performed the computational simulations within a broader range of period length. We randomly disturbed the parameter sets with different post-transcription time delays and manipulated the PLBS and ALBS (RORE) activity. As shown in Supplementary Figure S2, the monotonic relationship between the intensity ratio and the period length is not disrupted due to the disturbance of parameters and period ranges. This result indicates that if other oscillatory systems have a similar coupling structure in transcriptional level, the ratio rule may also exist. Furthermore, we can apply this topological structure in some synthetic oscillators, which requires certain period features. Therefore, these simulation results lead to a more general conclusion that it is the topological structure rather than the specified parameters served in this ratio-regulating pattern.

It should be noted that in this study we used the circadian period of locomotor assay and the mRNA expression in the liver to test our ratio hypothesis, and we do not exclude the hierarchical natures of the clock system between locomotor period and mRNA expression level in liver tissue. However, overall, a correlation between locomotor period and peripheral tissue period was seen in many studies (39,40). This correlation was especially striking in the period direction of short or long period. In this study, we are more concerned about the variation trend of the period length rather than the absolute value. Taken together, we speculated that the rule of the intensity ratio represents a general design principle underpinning the ratio-period regulating system.

The circadian clock can regulate the expression of multiple clock-controlled genes, and clock genes themselves contain many binding sites for clock-controlled genes in their promoters. Our results indicate that the combination of PER2-PPARα results in period alteration (Figure 5E). Although the underlying mechanism remains elusive, this information is of interest because more than half of nuclear receptors follow rhythmic cycles in key metabolic tissues (13), which has biological implications for the clock-receptor signal network. Some of these nuclear receptors may contribute to the circadian clock via other ALs. A recent genome-wide small interfering RNA-based study indicated that more than 200 genes play roles in clock biology by regulating clock amplitude and period (35), suggesting an unexpected level of reciprocal regulation between known signaling modules and the circadian network. This design principle provides multiple entry points to modulate the circadian clock to adapt to local environments. Meanwhile, the circadian clock should remain robust free-running period length due to its physiological role. Thus, there might be a trade-off between adaption and robustness. However, our study shows that variations in a single loop can only change the period length slightly. The intensity–balance property with the coupling effects in integrated loops provides a safeguard of the mammalian circadian system to maintain the period close to 24 h. Therefore, the circadian clock with multiple ALs can both meet the needs for adaptation and robustness.

This model not only facilitates the interpretation of results from existing experiments but also guides further experiments aimed at understanding the multiple interlocking wheels involved in clock coupling. Furthermore, it also provides an important clue for maintaining robustness in other biological regulatory networks with coupled loops.

## SUPPLEMENTARY DATA

Supplementary Data are available at NAR Online.

SUPPLEMENTARY DATA
