# Supplementary material for: An intensity ratio of interlocking loops determines circadian period length
Source: Nucleic Acids Res. 2014 Aug 13;42(16):10278–87. doi: 10.1093/nar/gku701 (PMC4176327; doi:10.1093/nar/gku701)
Supplement: SUPPLEMENTARY DATA [file supp_42_16_10278__index.html]

An intensity ratio of interlocking loops determines circadian period length — An intensity ratio of interlocking loops determines circadian period length — SUPPLEMENTARY DATA 

# An intensity ratio of interlocking loops determines circadian period length

## SUPPLEMENTARY DATA

**Files in this Data Supplement:**

- SUPPLEMENTARY DATA
