# Supplementary material for: An intensity ratio of interlocking loops determines circadian period length
Source: Nucleic Acids Res. 2014 Aug 13;42(16):10278–87. doi: 10.1093/nar/gku701 (PMC4176327; doi:10.1093/nar/gku701)
Supplement: SUPPLEMENTARY DATA [file supp_gku701_nar-01718-z-2014-File008.pdf]

Supplementary Information

1 Conceptual model

1.1 The construction of the conceptual model

In order to investigate how the auxiliary loops (ALs) coordinate with the negative primary loop to regulate the mammalian circadian clock, we firstly refine the core circadian network to a schematic model (Figure 1A and 1B) by ignoring all of the genetic redundancy. By comparing the negative primary feedback loop with and without a positive AL, we found that the AL can markedly contribute to the length of the period.

The regulatory scheme, depicted in Figure 1B, was used to construct the conceptual model. Here we did not describe the model of the primary loop-only scenario (Figure 1A) separately, as ,it can be obtained by setting the parameter, related to AL binding site (ALBS), to be zero.

The network in Figure 1B shows that: The expression of *R1* gene is enhanced by primary loop binding sites (PLBS); then, after a series of biochemical processes, the resulting R1 protein is translocated into the nucleus and binds to its own PLBS to repress PLBS mediated transcription. Thus, there is a negative primary feedback loop in the network (the pink rectangle box in Figure 1B). In addition, a positive auxiliary loop (AL) is formed: R1 protein binds to the PLBS of *R2* gene to inhibit the transcription of *R2*; in return, R2 protein feeds back to suppress the transcription of *R1* gene by binding to ALBS, which is another binding site in the regulation region of *R1* gene.

Then, we converted the diagram in Figure 1B into a set of equations. This mathematical model consists of the following molecular processes:

(1) The regulation of PLBS activity and ALBS activity

R1 protein binds to PLBS to inhibit the PLBS mediated transcription. Therefore, PLBS has two states: the active state without the repressor and the inactive state with the repressor. The probability of the active state is regarded as PLBS activity, which is denoted by $\mathrm{PLBS_{active}}$. In addition, the probability of the inactive state is denoted by $\mathrm{PLBS_{inactive}}$. In order to focus on the transcriptional regulation, we simply considered the post-translational duration, from the translation of R1 protein to R1 protein binding to PLBS in nucleus, as a fixed time τ_{P} in the mathematical model. Thus, the repressor binding to PLBS at present (at time t) depends on the cytoplasmic R1 protein in the past (at time $t-\tau_{\mathrm{P}}$). The repressor binding to PLBS is assumed to be dimerized by two R1 proteins. The process can be written as follows:

$$\mathrm{PLBS_{active}} \underset{\mathrm{k2}}{\overset{\mathrm{k1*R1^2}(t-\tau_{\mathrm{P}})}{\rightleftarrows}} \mathrm{PLBS_{inactive}},$$

$$\mathrm{PLBS_{active}} + \mathrm{PLBS_{inactive}} = 1,$$

$$\frac{d\mathrm{PLBS_{active}}}{dt} = -\mathrm{k1} * \mathrm{R1}^2(t-\tau_{\mathrm{P}}) * \mathrm{PLBS_{active}} + \mathrm{k2} * \mathrm{PLBS_{inactive}} \qquad (*).$$

To make the system simpler, PLBS activity was assumed to be set in quasi-steady state because the binding actions happen rapidly. Under this assumption, we can transform the differential equation (*) into an algebraic equation, as follows:

$$\mathrm{PLBS_{active}} * \mathrm{k1} * \mathrm{R1}^2(t-\tau_P) = \mathrm{k2} * (1-\mathrm{PLBS_{active}}),$$

Thus,

$$\mathrm{PLBS_{active}} = \frac{\mathrm{k2}}{\mathrm{k2} + \mathrm{k1} * \mathrm{R1}^2 (t - \tau_{\mathrm{P}})} \qquad (1.1').$$

R2 protein inhibits ALBS mediated transcription by binding to ALBS. Therefore, ALBS also has two states: the active state without nuclear R2 protein (denoted as $\mathrm{ALBS_{active}}$) and the inactive state with nuclear R2 protein (denoted as $\mathrm{ALBS_{inactive}}$). Similar to PLBS activity, the process can be written as follows:

$$\mathrm{ALBS_{active}} \underset{\mathrm{k4}}{\overset{\mathrm{k3*R2_N}}{\rightleftarrows}} \mathrm{ALBS_{inactive}},$$

$$\mathrm{ALBS_{active}} + \mathrm{ALBS_{inactive}} = 1,$$

$$\frac{d\mathrm{ALBS_{active}}}{dt} = -\mathrm{k3} * \mathrm{R2_N} * \mathrm{ALBS_{active}} + \mathrm{k4} * \mathrm{ALBS_{inactive}} \qquad (**).$$

R1 protein inhibits the transcription of *R2* gene through binding to its PLBS. For simplicity, we can assume that the nuclear R2 protein (denoted as $\mathrm{R2_N}$) is repressed by the cytoplasmic R1 protein at some time in the past ($t - \tau'$) directly. Therefore, the concentration of nuclear R2 can be expressed as a decreasing function of cytoplasmic R1 with another time delay τ'. The equation (**) can be written as follows:

$$\frac{d\mathrm{ALBS_{active}}}{dt} = -\mathrm{k3} * \frac{\mathrm{k2}}{\mathrm{k2} + \mathrm{k1} * \mathrm{R1}^2 (t - \tau')} * \mathrm{ALBS_{active}} + \mathrm{k4} * \mathrm{ALBS_{inactive}} \qquad (***)$$

Similar to PLBS activity, ALBS activity is also set in quasi-steady state, the equation $(***)$ is transferred into an algebraic equation:

$$\mathrm{ALBS_{active}} = \frac{\mathrm{k4}}{\mathrm{k4} + \mathrm{k3} * \dfrac{\mathrm{k2}}{\mathrm{k2} + \mathrm{k1} * \mathrm{R1}^2 (t - \tau')}} \qquad (1.2').$$

It's worthwhile to note that the primary loop in our model is a simplification of the E-box dominated negative feedback loops. The word "primary loop binding sites (PLBS)" in our model is basically the E-box, but with the phase delay due to other

cis-elements (for example, D-box). The effects are mainly reflected in the time delay τ_P. In this model, the phase of ALBS activity is assumed to be later than that of PLBS activity. It is similar to the relationship between RORE and the combination of E-box and D-box in the circadian clock system: the phase of RORE activity is about 8 hours later than that of the combination of E-box and D-box activity (1).

To explore how the positive AL coordinates with the negative primary loop to change the period, we added the constants K_P and K_A to the expressions of PLBS activity and ALBS activity to manipulate the oscillations of the primary loop and the auxiliary loop, respectively:

$$\mathrm{PLBS_{active}} = \mathrm{K_P} * \frac{\mathrm{k2}}{\mathrm{k2} + \mathrm{k1} * \mathrm{R1}^2 (t - \tau_\mathrm{P})} \qquad (1.1),$$

$$\mathrm{ALBS_{active}} = \mathrm{K_A} * \frac{\mathrm{k4}}{\mathrm{k4} + \mathrm{k3} * \mathrm{K_E} * \frac{\mathrm{k2}}{\mathrm{k2} + \mathrm{k1} * \mathrm{R1}^2 (t - \tau')}} \qquad (1.2).$$

When $\mathrm{K_A} = 0$, the expression of *R1* gene is only enhanced by PLBS, and the system has the negative primary loop only (Figure 1A).

(2) Transcription of *R1* gene

The expression of *R1* gene is dually mediated by PLBS and ALBS, and the transcription efficiencies enhanced by PLBS and ALBS in *R1* gene are proportional to PLBS activity and ALBS activity, respectively. We supposed that the transcriptions mediated by PLBS and ALBS are independent and do not interfere with each other. Therefore, the expression of *R1* mRNA (denoted as $mRNA$) can be expressed as a linear combination of PLBS activity and ALBS activity. In the mammalian circadian clock, it is validated that the expression of *Cry1* mRNA can be expressed by the linear

combination of cis-elements (1). Here, we have:

$$mRNA = \text{k\_P} * \text{PLBS}_{\text{activie}} + \text{k\_A} * \text{ALBS}_{\text{active}} \quad (1.3).$$

(3) Translation of *R1* mRNA

R1 mRNA translates into cytoplasmic protein (denoted as R1). The process is described by a differential equation. $\frac{d\text{R1}}{dt}$ is given by a simple kinetic equation, where the first term ($\text{ks} * mRNA$) is the rate of synthesis of cytoplasmic protein, and the second term $\text{kd} * \text{R1}$ is its rate of degradation. The variation of cytoplasmic R1 protein can be expressed as follow:

$$\frac{d\text{R1}}{dt} = \text{ks} * mRNA - \text{kd} * \text{R1} \quad (1.4).$$

(4) Post-translational modifications

The cytoplasmic R1 proteins go through a series of biochemical processes and form the dimer complexes to bind to the PLBS. Therefore, the concentration of the repressor that binds to PLBS is proportional to $\text{R1}^2(t - \tau_{\text{P}})$.

It is important to notice that we used τ_{P} to represent the post-transcriptional delay in the main text. It is because only the profiles of mRNA and the protein binding to the related *cis*-element (or nuclear protein) can be measured experimentally. In other words, it is the post-transcriptional delay, not the translational time delay, that can be observed in experiments. Because the difference between post-transcriptional time delay and post-translational time delay is close to a small constant, using post-transcriptional delay does not affect our results.

1.2 The numerical simulations

Based on these conditions, the conceptual model includes four equations from

equation (1.1) to equation (1.4). We summarized the values and the descriptions of the parameters in Table S1. Concentrations of this model are in arbitrary units.

After the construction of the model, we then performed the simulations to compare the conditions without and with the positive AL. We set $K_P = 1$ and $K_A = 0$ to mimic the primary loop-only situation (Figure 1A). In this situation, PLBS mediated transcription is only inhibited by R1 protein. Therefore, the simulation result in Figure 1C shows that the expression of *R1* gene is negatively related to R1 protein binding to PLBS, and the peak of *R1* mRNA is the trough of R1 protein binding to PLBS. The simulation result is normalized to $[0,1]$.

To introduce the positive AL to the system, we can change K_A from 0 to any positive constant to mimic the coordination of AL. Here, we used $K_A = 6.4$ to illustrate the role of the positive AL (Figure 1B).

Different from the primary loop-only system, the simulation result in Figure 1D shows that the positive AL can shift the phase of *R1* mRNA away from the trough of R1 binding to PLBS. Therefore, the positive auxiliary loop provides a transcriptional delay, τ_T, to confer the time delay of the negative primary loop. For comparison, the simulation result is also normalized to $[0,1]$.

Above numerical simulations are all done by MATLAB (Mathworks) with a solver for delayed differential equations (DDE23), and the integration step was 0.5. MATLAB code for the conceptual model (Figure 1B) is appended below.

```
clear all

global ks kd k1 k2 k3 k4 KP KA k_P k_A fid
```

```
hold on

k1=1;

k2=0.15;

k3=1;

k4=0.01;

KP=1;

KA=0;

ks=3;

kd=1;

k_P=1;

k_A=0.3;

lags=[10 4.5];

y0=[2];

fid=fopen('data.dat','W');

fprintf(fid,'t P R mRNA_R1 Repressor\n');

options = ddeset('Maxstep',0.5);

sol    = dde23(@fig1fun,lags,y0,[0,3100],options);

fclose(fid);

function dx=fig1fun(t,y,Z)

global    ks kd k1 k2 k3 k4 KP KA k_P k_A fid

R1=y(1);

PLBS=KP*k2/(k2+k1*Z(1,1)^2);
```

```
ALBS=KA*k4/(k4+k3*KP*k2/(k2+k1*Z(1,2)^2));

mRNA= k_P*PLBS+k_A*ALBS;

dR1_dt = ks*mRNA - kd*R1;

if t>3000

    fprintf(fid,'%16.8f %16.8f %16.8f %16.8f %16.8f\n',[t-3000 PLBS ALBS mRNA Z(1,1)]);

end

dx=[dR1_dt]';
```

Similar results can also be obtained in a comprehensive model with multiple genes. If we only consider the negative primary loop (The PERIOD and CRYPTOCHROME proteins can form the dimer complexes to inhibit their own transcription enhanced by PLBS), the trough of *Cry1* mRNA is the peak of repressors binding to PLBS (the peak of the comprehensive repression effect), and the peak of *Cry1* mRNA is the trough of repressors binding to PLBS (the trough of the comprehensive repression effect) (Figure S1A). When a positive AL is introduced in the system (CRY1 protein can inhibit the expression of *Rev-erb*α; in return, REV-ERBα binds to the RORE to inhibit the transcription of *Cry1*), a gap arises between the peak of *Cry1* mRNA and the trough of repressors binding to PLBS. Symmetrically, the trough of *Cry1* mRNA is also shifted away from the peak of repressors binding to PLBS (Figure S1B). The simulation result is normalized to $[0,1]$. The details of the comprehensive model are described in the following section of the comprehensive model.

Following the idea that the positive AL can separate the mRNA phase from the peak of PLBS activity in the conceptual model, we visualized this contribution of the positive auxiliary loop via experimental data. In the mammalian circadian clock, *Cry1* gene can also be enhanced by RORE (1). Therefore, a positive AL participates in the transcription of *Cry1* gene through RORE: CRY1 inhibits the transcription of *Rev-erbα* gene through binding to its E-box, and REV-ERBα feeds back to suppress the expression of *Cry1* gene through RORE. This structure is similar to that shown in Figure 1B. CRY1 protein and REV-ERBα protein correspond to R1 protein and R2 protein, separately. Thus, we measured the profiles of CRY1 binding to the E-box of the *Cry1* gene and histone deacetylase 3 (HDAC3) binding to the RORE of *Cry1* using chromatin immunoprecipitation (ChIP). HDAC3 has been shown to recruit REV-ERBα-containing complexes to the RORE and repress the transcription of Cry1 expression (2,3). *Cry1* mRNA was measured by using qPCR in mouse liver tissues to characterize the features of the separation of the repressors binding trough and the *Cry1* mRNA peak in the mammalian circadian clock. The data showed that the *Cry1* mRNA peak falls between the troughs of CRY1 and HDAC3 (Figure 1E). On the other side, the trough of *Cry1* mRNA is also between the peaks of CRY1 and the HDAC3. This experimental result suggested that the phase shift of *Cry1* mRNA is due to the participation of the AL. The profiles are fitted to sine function ($y_0 + A * \sin(\frac{\pi}{12}(x - x_c))$, where y_0, A, x_c are determined by experimental data) through the least square method. Therefore, the profile of CRY1 protein is fitted to

$$0.54035 + 0.27066 * \sin(\frac{\pi}{12}(x + 2.97)).$$

Furthermore, the expression of *Cry1* mRNA is fitted to

$$0.5423+0.3998*\sin(\frac{\pi}{12}(x-13.16842)).$$

To compare the experimental data with the simulation result, the fitted data were then normalized to $[0,1]$ (Figure 1F).

We further performed the numerical simulations by manipulating the amplitude of ALBS activity (by changing $\mathrm{K_A}$ from 0 to 6.5) and the amplitude of PLBS activity (by changing $\mathrm{K_P}$ from 0.28 to 1) to visually link the amplitudes of PLBS activity and ALBS activity to the period length. The simulation results show that the oscillation period is negatively related to $\frac{\mathrm{Amp_{PLBS}}}{\mathrm{Amp_{ALBS}}}$, and a single-activity amplitude is not enough to restrict the period (Figure 2).

We then performed the computational simulations within a broader range of period length to mimic other possible biological oscillators. The parameters (except τ_P and τ') in the deterministic model are multiplied by a stochastic factor, which was randomly chosen from uniform distribution (from 0.5 to 1.5). After that, we manipulated the amplitude of ALBS activity (by changing $\mathrm{K_A}$ from 0 to 6.5) and the amplitude of PLBS activity (by changing $\mathrm{K_P}$ from 0.28 to 1) to observe the period variations. The simulations are performed in ten parameter sets and Figure S2A exhibits two typical results. As seen from Figure S2A, the period length is still reversely related to the amplitude ratio even when the parameters are changed. We then performed the same simulations in the situations when $\tau_P=8$, $\tau'=3.5$; $\tau_P=6$, $\tau'=2.5$. Since the negative feedback loop need a critical time delay to remain the oscillation, the period of the oscillation has a minimal length. As shown in Figure

S2B-C, the period length can be reduced to ~14h and the monotonic relationship between the intensity ratio and the period length is not disrupted. Then we gradually increased the time delays and tried the situations when $\tau_P = 12$, $\tau' = 5.5$; $\tau_P = 14$, $\tau' = 6.5$; $\tau_P = 20$, $\tau' = 9.5$; $\tau_P = 30$, $\tau' = 14.5$; $\tau_P = 40$, $\tau' = 19.5$; $\tau_P = 50$, $\tau' = 24.5$; $\tau_P = 60$, $\tau' = 29.5$. Similarly, the monotonic relationship between the intensity ratio and the period length still remains and the period can increased to ~148h (Figure S2D-J). This result indicates that if other oscillatory systems with different period ranges have the similar coupling structure in transcriptional level, the ratio rule may also exist.

These computational simulations are implemented by using FORTRAN 95, and the integration step was 0.05.

1.3 The theoretical estimation

We also used the theoretical analysis to estimate the transcriptional time delay τ_{T}. In the theoretical analysis, we assumed that the oscillations of the PLBS activity and ALBS activity are cosine waves. The expression of ALBS activity is assumed to be later than that of PLBS activity, as mentioned above.

We used $\mathrm{E}(t)$ and $\mathrm{ALBS}(t)$ to represent PLBS activity and ALBS activity at time t respectively, which are defined as:

$$\mathrm{P}(t) = \mathrm{P}_0 + \mathrm{Amp}_{\mathrm{PLBS}} \cos(\frac{2\pi}{\alpha}(t - \tau_{\mathrm{PLBS}})),$$

$$\mathrm{ALBS}(t) = \mathrm{ALBS}_0 + \mathrm{Amp}_{\mathrm{ALBS}} \cos(\frac{2\pi}{\alpha}(t - \tau_{\mathrm{ALBS}})),$$

$$0 \le \mathrm{P}(t) \le 1,$$

$0 \leq \mathrm{ALBS}(t) \leq 1$,

$0 < \tau_{\mathrm{PLBS}} < \tau_{\mathrm{ALBS}} < \frac{1}{2}\alpha$.

where $\mathrm{Amp}_{\mathrm{PLBS}}$ and $\mathrm{Amp}_{\mathrm{ALBS}}$ are the amplitudes, τ_{PLBS} and τ_{ALBS} are the phases of PLBS activity and ALBS activity. P_0 and ALBS_0 are constants. α represents the period of the oscillation.

According to Figure 1B, the expression of *R1* gene is dually enhanced by PLBS and ALBS. Similar to the above conceptual model, the expression of *R1* mRNA at time t, which is denoted as $mRNA(t)$, can be regarded as the linear combination of PLBS activity and ALBS activity:

$$\begin{aligned}
mRNA(t) &= \mathrm{k_P} * \mathrm{P}(t) + \mathrm{k_A} * \mathrm{ALBS}(t) \\
&= \mathrm{k_P} * (\mathrm{E}_0 + \mathrm{Amp}_{\mathrm{PLBS}} \cos(\frac{2\pi}{\alpha}(t - \tau_{\mathrm{PLBS}}))) \\
&\quad + \mathrm{k_A} * (\mathrm{ALBS}_0 + \mathrm{Amp}_{\mathrm{ALBS}} \cos(\frac{2\pi}{\alpha}(t - \tau_{\mathrm{ALBS}}))) \\
&= \mathrm{k_P} * \mathrm{P}_0 + \mathrm{k_A} * \mathrm{ALBS}_0 \\
&\quad + \sqrt{M^2 + N^2}\,(\frac{M}{\sqrt{M^2 + N^2}} \cos(\frac{2\pi}{\alpha}t) + \frac{N}{\sqrt{M^2 + N^2}} \sin(\frac{2\pi}{\alpha}t)) \\
&= \mathrm{k_P} * \mathrm{P}_0 + \mathrm{k_A} * \mathrm{ALBS}_0 + \sqrt{M^2 + N^2} \cos(\frac{2\pi}{\alpha}(t - \varphi^*))
\end{aligned},$$

where

$\varphi^* \in [0, \frac{1}{2}\alpha]$,

$M = \mathrm{k\_P} * \mathrm{Amp}_{\mathrm{PLBS}} \cos(\frac{2\pi}{\alpha}\tau_{\mathrm{PLBS}}) + \mathrm{k\_A} * \mathrm{Amp}_{\mathrm{ALBS}} \cos(\frac{2\pi}{\alpha}\tau_{\mathrm{ALBS}})$,

$N = \mathrm{k\_P} * \mathrm{Amp}_{\mathrm{PLBS}} \sin(\frac{2\pi}{\alpha}\tau_{\mathrm{PLBS}}) + \mathrm{k\_A} * \mathrm{Amp}_{\mathrm{ALBS}} \sin(\frac{2\pi}{\alpha}\tau_{\mathrm{ALBS}})$,

and

$\cos(\frac{2\pi}{\alpha}\varphi^*) = \frac{M}{\sqrt{M^2 + N^2}}$.

The expression of φ^* can be derived as follows,

$$\cos(\frac{2\pi}{\alpha}\varphi^*) = \frac{p\frac{\mathrm{Amp}_{\mathrm{PLBS}}}{\mathrm{Amp}_{\mathrm{ALBS}}}+q}{\sqrt{(p\frac{\mathrm{Amp}_{\mathrm{PLBS}}}{\mathrm{Amp}_{\mathrm{ALBS}}}+q)^2+(m\frac{\mathrm{Amp}_{\mathrm{PLBS}}}{\mathrm{Amp}_{\mathrm{ALBS}}}+n)^2}}$$

where m, n, p, q are all constants,

$p = \mathrm{k\_P} * \cos(\frac{2\pi}{\alpha}\tau_{\mathrm{PLBS}})$,

$q = \mathrm{k\_A} * \cos(\frac{2\pi}{\alpha}\tau_{\mathrm{ALBS}})$,

$m = \mathrm{k\_P} * \sin(\frac{2\pi}{\alpha}\tau_{\mathrm{PLBS}})$,

$n = \mathrm{k\_A} * \sin(\frac{2\pi}{\alpha}\tau_{\mathrm{ALBS}})$.

If $p\frac{\mathrm{Amp}_{\mathrm{PLBS}}}{\mathrm{Amp}_{\mathrm{ALBS}}}+q \geq o$,

$\cos(\frac{2\pi}{\alpha}\varphi^*) = \frac{1}{\sqrt{1+\left(\frac{m\frac{\mathrm{Amp}_{\mathrm{PLBS}}}{\mathrm{Amp}_{\mathrm{ALBS}}}+n}{p\frac{\mathrm{Amp}_{\mathrm{PLBS}}}{\mathrm{Amp}_{\mathrm{ALBS}}}+q}\right)^2}}$. $\cos(\frac{2\pi}{\alpha}\varphi^*)$ can increase as $\frac{\mathrm{Amp}_{\mathrm{PLBS}}}{\mathrm{Amp}_{\mathrm{ALBS}}}$ increase. Therefore φ^* is negatively related to $\frac{\mathrm{Amp}_{\mathrm{PLBS}}}{\mathrm{Amp}_{\mathrm{ALBS}}}$.

If $p\frac{\mathrm{Amp}_{\mathrm{PLBS}}}{\mathrm{Amp}_{\mathrm{ALBS}}}+q < o$,

$\cos(\frac{2\pi}{\alpha}\varphi^*) = \frac{-1}{\sqrt{1+\left(\frac{m\frac{\mathrm{Amp}_{\mathrm{PLBS}}}{\mathrm{Amp}_{\mathrm{ALBS}}}+n}{-p\frac{\mathrm{Amp}_{\mathrm{PLBS}}}{\mathrm{Amp}_{\mathrm{ALBS}}}-q}\right)^2}}$. $\cos(\frac{2\pi}{\alpha}\varphi^*)$ can increase as $\frac{\mathrm{Amp}_{\mathrm{PLBS}}}{\mathrm{Amp}_{\mathrm{ALBS}}}$ increase. Therefore φ^* is negatively related to $\frac{\mathrm{Amp}_{\mathrm{PLBS}}}{\mathrm{Amp}_{\mathrm{ALBS}}}$. Here, φ^* is the phase of *R1* mRNA; therefore, the transcriptional time delay τ_{T} is:

$\tau_{\mathrm{T}} = \varphi^* - \tau_{\mathrm{PLBS}}$, where τ_{PLBS} is a constant.

In summary, the transcriptional delay τ_T is the function of the amplitude ratio between PLBS activity and ALBS activity: $\tau_T = f(\frac{\text{Amp}_{\text{PLBS}}}{\text{Amp}_{\text{ALBS}}})$. Furthermore, the transcriptional delay τ_T increases with the decrease of $\frac{\text{Amp}_{\text{PLBS}}}{\text{Amp}_{\text{ALBS}}}$.

2 Comprehensive model

2.1 The construction of the comprehensive model

We next investigated whether the relationship between the amplitude ratio and the period, which emerges from the conceptual model, also exists in a real circadian network with much more complexity. The mammalian circadian system also consists of a negative feedback loop and a positive feedback loop: *Per* and *Cry* genes are involved in the negative primary loop (4,5). On the other side, CRY1 can inhibit the expression of *Rev-erbα* gene; in return, REV-ERBα can bind to the RORE of *Cry1* gene to suppress its expression level (1). Thus, a positive AL is formed.

Then, we constructed a comprehensive model with 6 genes (*Bmal1*, *Per1*, *Cry1*, *Per2*, *Cry2*, and *Rev-erbα*) to confirm the above ratio hypothesis. In order to focus on the transcriptional regulations, we assumed that the post-translational time delay of each gene is fixed as an explicit time delay. The schematic view of the comprehensive model is exhibited in Figure S3. The comprehensive model includes the following process:

(1) The regulations of PLBS activity and RORE activity

Similar to the conceptual model, there are two states for PLBS and RORE: the active state (denoted as $\text{PLBS}_{\text{active}}$ and $\text{RORE}_{\text{active}}$, respectively) and the inactive

state (denoted as $\mathrm{PLBS_{inactive}}$ and $\mathrm{RORE_{inactive}}$, respectively). Besides the basic transcription, only the active state can enhance the transcription of the genes. The active state of PLBS is promoted by nuclear BMAL1 (denoted as $\mathrm{BMAL1_N}$), and it is suppressed by nuclear PER1:CRY1 (denoted as $\mathrm{P1C1}$), PER1:CRY2 (denoted as $\mathrm{P1C2}$), PER2:CRY1 (denoted as $\mathrm{P2C1}$), PER2:CRY2 (denoted as $\mathrm{P2C2}$) complexes. The comprehensive repression effect (denoted by I) on the PLBS are the sum of these nuclear dimer complexes with different binding coefficients (α_1, α_2, α_3, α_4). Therefore, the regulation of PLBS activity is described as follows:

$$\mathrm{PLBS_{active}} \underset{\mathrm{k1*BMAL1_N}}{\overset{\mathrm{I}}{\rightleftarrows}} \mathrm{PLBS_{inactive}},$$

$$\mathrm{PLBS_{active}} + \mathrm{PLBS_{inactive}} = 1,$$

$$\frac{d\mathrm{PLBS_{active}}}{dt} = -\mathrm{PLBS_{active}} * \mathrm{I} + \mathrm{k1} * \mathrm{BMAL1_N} * \mathrm{PLBS_{inactive}},$$

$$\mathrm{I} = \alpha_1 * \mathrm{P1C1} + \alpha_2 * \mathrm{P1C2} + \alpha_3 * \mathrm{P2C1} + \alpha_4 * \mathrm{P2C2}.$$

The active state of RORE is repressed by nuclear RER-ERBα (denoted as $\mathrm{REV_N}$). The regulation of RORE activity is described as follows:

$$\mathrm{RORE_{active}} \underset{\mathrm{k2}}{\overset{\beta * \mathrm{REV_N}}{\rightleftarrows}} \mathrm{RORE_{inactive}},$$

$$\mathrm{RORE_{active}} + \mathrm{RORE_{inactive}} = 1,$$

$$\frac{d\mathrm{RORE_{active}}}{dt} = \mathrm{k2} * \mathrm{RORE_{inactive}} - \beta * \mathrm{REV_N} * \mathrm{RORE_{active}}.$$

Similar to the conceptual model, PLBS activity and RORE activity are in quasi-steady state because of the instant reactions. Therefore, the activity of PLBS and RORE can be expressed as algebraic equations:

$$\mathrm{PLBS_{active}} = \frac{\mathrm{k1} * \mathrm{BMAL1_N}}{\mathrm{k1} * \mathrm{BMAL1_N} + \mathrm{I}} \qquad (2.1),$$

$$\mathrm{RORE_{active}} = \frac{\mathrm{k2}}{\mathrm{k2} + \beta * \mathrm{REV_N}} \qquad (2.2),$$

where

$$\mathrm{I} = \alpha_1 * \mathrm{P1C1} + \alpha_2 * \mathrm{P1C2} + \alpha_3 * \mathrm{P2C1} + \alpha_4 * \mathrm{P2C2}.$$

(2) Transcriptions of *Per1, Per2, Cry1, Cry2, Bmal1, Rev-erb*α genes

Per1 mRNA, *Per2* mRNA, *Cry1* mRNA, *Cry2* mRNA, *Bmal1* mRNA, *Rev-erb*α mRNA are denoted as $Per1$, $Per2$, $Cry1$, $Cry2$, $Bmal1$, and Rev, respectively. We assumed that the expressions of *Per1*, *Per2, Cry2 and Rev-erb*α are all enhanced by PLBS, the expression of *Bmal1* is enhanced by RORE activity, and the transcriptions of *Cry1* gene is enhanced by both PLBS and RORE (5). The RORE activity is also later than that of PLBS, so the expression of *Cry1* can be delayed. It's notable that the experimental data shows that the phase of *Per2* mRNA is at about CT 12~16, which is later than that of *Per1* mRNA (CT 12). Also, *Rev-erb*α mRNA peaks at CT 4, which is earlier than that of *Per1* mRNA. Therefore, besides PLBS, there might be some other factors can regulate the phase of *Per2* mRNA and *Rev-erb*α mRNA. To focus on the coordination of the negative and the positive RORE feedback loops, we respectively simplified the resultant phases of these unclear regulations in *Per2* mRNA and *Rev-erb*α mRNA as explicit time delays, τ_{per2} and τ_{rev}. Thus, the transcriptions of these genes can be expressed as follows:

$$Bmal1 = \mathrm{kb} * \frac{(\mathrm{k\_ROREb} * \mathrm{RORE_{active}})^2}{(\mathrm{k\_ROREb} * \mathrm{RORE_{active}})^2 + \mathrm{K\_Bmal1}^2} + \mathrm{v_{0b}} \qquad (2.3),$$

$$Per1 = \mathrm{kp} * \frac{(\mathrm{k\_PLBSp} * \mathrm{PLBS_{active}})^2}{(\mathrm{k\_PLBSp} * \mathrm{PLBS_{active}})^2 + \mathrm{K\_Per}^2} + \mathrm{v_{0p}} \qquad (2.4),$$

$$Cry1 = \mathrm{kc} * \frac{(\mathrm{k\_PLBSc} * \mathrm{PLBS}_{\mathrm{active}} + \mathrm{k\_ROREc} * \mathrm{RORE}_{\mathrm{active}})^2}{(\mathrm{k\_PLBSc} * \mathrm{PLBS}_{\mathrm{active}} + \mathrm{k\_ROREc} * \mathrm{RORE}_{\mathrm{active}})^2 + \mathrm{K\_Cry}^2} + \mathrm{v}_{0\mathrm{c}} \quad (2.5),$$

$$Per2 = \mathrm{kp2} * \frac{(\mathrm{k\_PLBSp2} * \mathrm{PLBS}_{\mathrm{active}}(t - \tau_{Per2}))^2}{(\mathrm{k\_PLBSp2} * \mathrm{PLBS}_{\mathrm{active}}(t - \tau_{Per2}))^2 + \mathrm{K\_Per2}^2} + \mathrm{v}_{0\mathrm{p2}} \quad (2.6),$$

$$Cry2 = \mathrm{kc2} * \frac{(\mathrm{k\_PLBSc2} * \mathrm{PLBS}_{\mathrm{active}})^2}{(\mathrm{k\_PLBSc2} * \mathrm{PLBS}_{\mathrm{active}})^2 + \mathrm{K\_Cry2}^2} + \mathrm{v}_{0\mathrm{c2}} \quad (2.7),$$

$$Rev = kr * \frac{(k\_PLBSr * PLBS_{active}(t - \tau_{Rev}))^2}{(k\_PLBSr * PLBS_{active}(t - \tau_{Rev}))^2 + K\_Rev^2} + v_{0r} \quad (2.8).$$

(3) Translation of *Per1* mRNA, *Per2* mRNA, *Cry1* mRNA, *Cry2* mRNA, *Bmal1* mRNA, *Rev-erb*α mRNA

The cytoplasmic PER1, PER2, CRY1, CRY2, BMAL1 and REV-ERBα proteins are denoted as $\mathrm{PER_C}$, $\mathrm{PER2_C}$, $\mathrm{CRY_C}$, $\mathrm{CRY2_C}$, $\mathrm{BMAL1_C}$, and $\mathrm{REV_C}$, respectively. The processes are described by differential equations:

$$\frac{d\mathrm{BMAL1_C}}{dt} = \mathrm{V\_Bmal1} * Bmal1 - \mathrm{kd\_Bmal1c} * \mathrm{BMAL1_C} \quad (2.9),$$

$$\frac{d\mathrm{PER_C}}{dt} = \mathrm{V\_Per} * Per1 - \mathrm{kd\_Perc} * \mathrm{PER_C} \quad (2.10),$$

$$\frac{d\mathrm{CRY_C}}{dt} = \mathrm{V\_Cry} * Cry1 - \mathrm{kd\_Cryc} * \mathrm{CRY_C} \quad (2.11),$$

$$\frac{d\mathrm{PER2_C}}{dt} = \mathrm{V\_Per2} * Per2 - \mathrm{kd\_Per\,2c} * \mathrm{PER2_C} \quad (2.12),$$

$$\frac{d\mathrm{CRY2_C}}{dt} = \mathrm{V\_Cry2} * Cry2 - \mathrm{kd\_Cry2c} * \mathrm{CRY2_C} \quad (2.13),$$

$$\frac{d\mathrm{REV_C}}{dt} = \mathrm{V\_Rev} * Rev - \mathrm{kd\_Revc} * \mathrm{REV_C} \quad (2.14).$$

(4) The post-translational regulations

To focus on the transcriptional regulations, we assumed that the

post-translational time delays of cytoplasmic PER1, PER2, CRY1, CRY2, BMAL1 and REV-ERBα are fixed as τ_1, τ_2, τ_3, τ_4, τ_5, and τ_6, respectively. Therefore, the nuclear BMAL1 (denoted as $\mathrm{BMAL1_N}$) is expressed by cytoplasmic BMAL1 τ_1 hours ago ($\mathrm{BMAL1_C}(t-\tau_1)$), and the nuclear REV-ERBα (denoted as $\mathrm{REV_N}$) is expressed by cytoplasmic REV-ERBα τ_6 hours ago ($\mathrm{REV_C}(t-\tau_6)$). The nuclear PER1:CRY1 complex (denoted as P1C1) is determined by the cytoplasmic PER1 protein τ_2 h ago and the cytoplasmic CRY1 protein τ_4 h ago. Similarly, the concentrations of P1C2, P2C1, and P2C2 are all determined by the corresponding cytoplasmic proteins at some time in the past. The post-translational time delays are estimated by experimental data.

Therefore, these nuclear proteins can be written as follows:

$$\mathrm{BMAL1_N} = \mathrm{K_{Bmal1}} * \mathrm{BMAL1_C}(t-\tau_1) \qquad (2.15),$$

$$\mathrm{REV_N} = \mathrm{K_{Rev}} * \mathrm{REV_C}(t-\tau_6) \qquad (2.16),$$

$$\frac{d\mathrm{P1C1}}{dt} = \mathrm{ks\_p1c1} * \mathrm{PER_C}(t-\tau_2) * \mathrm{CRY_C}(t-\tau_4) - \mathrm{kd\_P1C1} * \mathrm{P1C1} \qquad (2.17),$$

$$\frac{d\mathrm{P1C2}}{dt} = \mathrm{ks\_p1c2} * \mathrm{PER_C}(t-\tau_2) * \mathrm{CRY2_C}(t-\tau_5) - \mathrm{kd\_P1C2} * \mathrm{P1C2} \qquad (2.18),$$

$$\frac{d\mathrm{P2C1}}{dt} = \mathrm{ks\_p2c1} * \mathrm{PER2_C}(t-\tau_3) * \mathrm{CRY_C}(t-\tau_4) - \mathrm{kd\_P2C1} * \mathrm{P2C1} \qquad (2.19),$$

$$\frac{d\mathrm{P2C2}}{dt} = \mathrm{ks\_p2c2} * \mathrm{PER2_C}(t-\tau_3) * \mathrm{CRY2_C}(t-\tau_5) - \mathrm{kd\_P2C2} * \mathrm{P2C2} \qquad (2.20).$$

2.2 The numerical simulations

Under the above conditions, we can convert the mammalian circadian regulation network into a set of delay differential equations and algebraic equations, from

equation (2.1) to equation (2.20), which include 10 variations. During the exploration of the model parameters, we found that the post-translational time delays are the main factors, which significantly change the period of the oscillation. Therefore, the time delay of each gene is well estimated from the experimental data. Because other parameters do not significantly affect the period, we chose these parameters in a proper range after we made reference to previous modeling works (6-12). The value and the description of each parameter are listed in Table S2. Concentrations of this model are in arbitrary units. We then changed all the parameters in WT case from 70% to 130% to observe the effect of each parameter to the period length. The result is also summarized in Table S2, which suggests that the period length is more sensitive to the post-transcriptional time delay of the repressor genes.

The numerical simulation is performed by MATLAB (Mathworks) with a solver for delayed differential equations (DDE23), and the integration step was 0.5. MATLAB code for the comprehensive model (WT case) is appended below.

```
clear all
global v0p v0p2 v0c v0c2 v0b v0r ks_p1c1 ks_p1c2 ks_p2c1 ks_p2c2 kd_p1c1 kd_p1c2 kd_p2c1
kd_p2c2 kb kp kc kp2 kc2 kr K_Bmal1 K_Per K_Per2 K_Cry K_Cry2 K_Rev k_PLBSp k_PLBSp2
k_PLBSc k_ROREc k_PLBSc2 k_PLBSr k_ROREr k_ROREb V_Bmal1 V_Per V_Per2 V_Cry
V_Cry2 V_Rev kd_Bmal1c kd_Perc kd_Per2c kd_Cryc kd_Cry2c kd_Revc Kbmal1 Krev k1 k2
alpha1 alpha2 alpha3 alpha4 beta fid
hold on
```

k1=0.1;

k2=10;

k_PLBSp=1;

kp=10000;

V_Per=7;

kd_Perc=1.8;

k_PLBSp2=1;

kp2=10000;

V_Per2=8;

kd_Per2c=1.8;

k_PLBSc2=1;

kc2=10000;

V_Cry2=5;

kd_Cry2c=1.8;

k_PLBSc=0.6;

k_ROREc=0.8;

kc=10000;

V_Cry=7;

kd_Cryc=1.8;

k_PLBSr=1;

kr=10000;

V_Rev=10;

kd_Revc=1;

k_ROREb=1;

kb=12;

V_Bmal1=0.1;

kd_Bmal1c=0.5;

ks_p1c1=1.8;

kd_p1c1=1.25;

ks_p2c1=1.5;

kd_p2c1=1.4;

ks_p1c2=2;

kd_p1c2=2;

ks_p2c2=1;

kd_p2c2=2;

v0p=0.01;

v0p2=0.056;

v0c=0.01;

v0c2=0.4;

v0b=8;

v0r=0.0001;

K_Bmal1=0.1;

K_Per=100;

K_Per2=100;

```
K_Cry=100;

K_Cry2=100;

K_Rev=100;

Kbmal1=1;

Krev=1;

alpha1=0.75;

alpha2=0.15;

alpha3=0.075;

alpha4=0.3;

beta=5;

lags=[12 10 10.8 9.8 9.8 21];

y0=[19 3 3 3 3 3 3 2 2 3];

fid=fopen('primary.dat','W');

fprintf(fid, 't Per1 Per2 Cry1 Cry2 PERc PER2c CRYc CRY2c PLBS RRE Repressor\n');

options = ddeset('Maxstep',0.5);

sol    = dde23(@WTfun,lags,y0,[0,3100],options);

fclose(fid);


function dy=WTfun(t,y,Z)

global v0p v0p2 v0c v0c2 v0b v0r ks_p1c1 ks_p1c2 ks_p2c1 ks_p2c2 kd_p1c1 kd_p1c2 kd_p2c1

kd_p2c2 kb kp kc kp2 kc2 kr K_Bmal1 K_Per K_Per2 K_Cry K_Cry2 K_Rev k_PLBSp k_PLBSp2

k_PLBSc k_ROREc k_PLBSc2 k_PLBSr k_ROREr k_ROREb V_Bmal1 V_Per V_Per2 V_Cry
```

```
V_Cry2 V_Rev Kbmal1 Krev kd_Bmal1c kd_Perc kd_Per2c kd_Cryc kd_Cry2c kd_Revc k1 k2
alpha1 alpha2 alpha3 alpha4 beta fid
BMAL1c=y(1);
PERc=y(2);
PER2c=y(3);
CRYc=y(4);
CRY2c=y(5);
REVc=y(6);
P1C1=y(7);
P1C2=y(8);
P2C1=y(9);
P2C2=y(10);
BMALlN=Kbmal1*Z(1,1);
Per_tau2=Z(2,2);
Per2_tau3=Z(3,3);
Cry_tau4=Z(4,4);
Cry2_tau5=Z(5,5);
REVN=Krev*Z(6,6);
PLBS=(BMALlN*k1)/(P1C1*alpha1+P1C2*alpha2+P2C1*alpha3+P2C2*alpha4+BMALlN*k1);
RORE=k2/(k2+REVN*beta);
Bmal1=kb*(k_ROREb*RORE)^2/(K_Bmal1^2+(k_ROREb*RORE)^2)+v0b;
dBMAL1c_dt=V_Bmal1*Bmal1-kd_Bmal1c*BMAL1c;
```

```
Per1=kp*(k_PLBSp*PLBS)^2/(K_Per^2+(k_PLBSp*PLBS)^2)+v0p;

dPERc_dt=V_Per*Per1-kd_Perc*PERc;

Cry1=kc*(k_PLBSc*PLBS+k_ROREc*RORE)^2/(K_Cry^2+(k_PLBSc*PLBS+k_ROREc*RORE)^2
)+v0c;

dCRYc_dt=V_Cry*Cry1-kd_Cryc*CRYc;

Per2=kp2*(k_PLBSp2*PLBS)^2/(K_Per2^2+(k_PLBSp2*PLBS)^2)+v0p2;

dPER2c_dt=V_Per2*Per2-kd_Per2c*PER2c;

Cry2=kc2*(k_PLBSc2*PLBS)^2/(K_Cry2^2+(k_PLBSc*PLBS)^2)+v0c2;

dCRY2c_dt=V_Cry2*Cry2-kd_Cry2c*CRY2c;

dP1C1_dt=ks_p1c1*Per_tau2*Cry_tau4-kd_p1c1*P1C1;

dP1C2_dt=ks_p1c2*Per_tau2*Cry2_tau5-kd_p1c2*P1C2;

dP2C1_dt=ks_p2c1*Per2_tau3*Cry_tau4-kd_p2c1*P2C1;

dP2C2_dt=ks_p2c2*Per2_tau3*Cry2_tau5-kd_p2c2*P2C2;

Rev=kr*(k_PLBSr*PLBS)^2/(K_Rev^2+(k_PLBSr*PLBS)^2)+v0r;

dREVc_dt=V_Rev*Rev-kd_Revc*REVc;

if t>3000


fprintf(fid,'%16.8f %16.8f %16.8f %16.8f %16.8f %16.8f %16.8f %16.8f %16.8f %16.8f %16.8f %16.
8f\n',[t-3000 Per1 Per2 Cry1 Cry2 PERc PER2c CRYc CRY2c PLBS RORE
P1C1*alpha1+P1C2*alpha2+P2C1*alpha3+P2C2*alpha4]);

end

dy=[dBMAL1c_dt dPERc_dt dPER2c_dt dCRYc_dt dCRY2c_dt dREVc_dt dP1C1_dt dP1C2_dt
```

dP2C1_dt dP2C2_dt]';

To check whether the model is reliable, we used the model to perform numerical simulations under different conditions. The periods in the simulations are compared with the experimental data. The results are listed in Table S3. Also, the comprehensive model places the expressions of the genes approximately in-phase in WT case (Table S4). Therefore, we believe that this validated model can provide a qualitatively predictive tool for studying the properties of the circadian clock. Then, we used the model to check the relationship between the amplitude ratio $\frac{\mathrm{Amp}_{\mathrm{PLBS}}}{\mathrm{Amp}_{\mathrm{RORE}}}$ and the period of the oscillation.

After the reliability of the model is confirmed, we performed the simulations with different amplitudes of PLBS activity (denoted as $\mathrm{Amp}_{\mathrm{PLBS}}$) and RORE activity (denoted as $\mathrm{Amp}_{\mathrm{RORE}}$) to check if the comprehensive model also fit the ratio hypothesis. We got different $\mathrm{Amp}_{\mathrm{PLBS}}$ and $\mathrm{Amp}_{\mathrm{RORE}}$ by varying the parameters which represent the binding coefficients of the repressors to PLBS (α_i, $i = 1, 2, 3, 4$) and RORE (β). α_i are changed from $0.5 * \alpha_i$ to $9.5 * \alpha_i$, and β is changed from $0.08 * \beta$ to $4.04 * \beta$.

The numerical simulation is presented in Figure 3A-C, which also supports the hypothesis that the amplitude ratio $\frac{\mathrm{Amp}_{\mathrm{PLBS}}}{\mathrm{Amp}_{\mathrm{RORE}}}$ is negatively related to the period of the oscillation. However, neither $\mathrm{Amp}_{\mathrm{PLBS}}$ nor $\mathrm{Amp}_{\mathrm{RORE}}$ has any clear monotonic relevance with the period (Figure 3B and 3C).

In the integrated loops, the activities of PLBS and RORE are partially associated

with each other. Therefore, there is a coupling effect between the loops which can help to ensure period robustness. To investigate this coupling effect in the system, we exhibited how the amplitude of RORE activity is varied as changes only occur in the inhibition strength of PLBS. The resultant amplitude variation of PLBS activity can be completely passed on to the amplitude of RORE activity (α_i are changed from $0.5 * \alpha_i$ to $9.5 * \alpha_i$, Figure 4A). Correspondently, when changes only occur in the inhibition of RORE, the variation in the amplitude of RORE activity can be partially transmitted to the amplitude of PLBS activity (β is changed from $0.6 * \beta$ to $4.04 * \beta$, Figure 4B). The amplitude variations of PLBS and RORE are defined as the ratios of the resultant amplitude to the WT case. Note that although the inhibitor strengths can be largely changed, the amplitude variations are limited in a narrow range. We further present the relationship between the period and the relative inhibition strength to binding elements (Figure 4C) in three-dimensional space. The binding coefficients α_i are changed from $0.5 * \alpha_i$ to $4.7 * \alpha_i$, and β is changed from $0.6 * \beta$ to $4.04 * \beta$. The vertical axis represents the period length, and the two horizontal axes represent the individual strengths of the inhibition of the PLBS and RORE. The period of the oscillation remains approximately constant (the yellow line) when the change is only applied to the inhibition of PLBS. When this inhibition strength is increased to 4.7 times, the period is only changed to 24.35 h (the cyan bar). Similarly, the period is slightly changed (the black line) if the inhibition variation only occurs to RORE, and even this inhibition strength is increased to 4.04 times, the period is changed to 23.5 h (the purple bar). Compare to the period of the WT (the

yellow bar, 24.02 h), changing the inhibition on one loop does not effectively alter the period. The simulation result also shows that if both inhibitions of PLBS and RORE increase, the period will significantly raise along a ridge (the red dash line) (Figure 4C). The period will reach 26.35h when the inhibition strengths to PLBS and RORE are increased to 4.7 times and 4.04 times respectively (the red bar).

These computational simulations in comprehensive model are done by using FORTRAN 95, and the integration step was 0.05.

References

1. Ukai-Tadenuma, M., Yamada, R.G., Xu, H., Ripperger, J.A., Liu, A.C. and Ueda, H.R. (2011) Delay in feedback repression by cryptochrome 1 is required for circadian clock function. *Cell*, **144**, 268-281.
2. Yin, L. and Lazar, M.A. (2005) The Orphan Nuclear Receptor Rev-erb{alpha} Recruits the N-CoR/Histone Deacetylase 3 Corepressor to Regulate the Circadian Bmal1 Gene. *Mol Endocrinol*, **19**, 1452-1459.
3. Alenghat, T., Meyers, K., Mullican, S.E., Leitner, K., Adeniji-Adele, A., Avila, J., Bucan, M., Ahima, R.S., Kaestner, K.H. and Lazar, M.A. (2008) Nuclear receptor corepressor and histone deacetylase 3 govern circadian metabolic physiology. *Nature*, **456**, 997-1000.
4. Gallego, M. and Virshup, D.M. (2007) Post-translational modifications regulate the ticking of the circadian clock. *Nature reviews. Molecular cell biology*, **8**, 139-148.
5. Ueda, H.R., Hayashi, S., Chen, W., Sano, M., Machida, M., Shigeyoshi, Y., Iino, M. and Hashimoto, S. (2005) System-level identification of transcriptional circuits underlying mammalian circadian clocks. *Nature genetics*, **37**, 187-192.
6. Goldbeter, A. (1995) A model for circadian oscillations in the Drosophila period protein (PER). *Proceedings. Biological sciences / The Royal Society*, **261**, 319-324.
7. Leloup, J.C. and Goldbeter, A. (2003) Toward a detailed computational model for the mammalian circadian clock. *Proceedings of the National Academy of Sciences of the United States of America*, **100**, 7051-7056.
8. Forger, D.B. and Peskin, C.S. (2003) A detailed predictive model of the mammalian circadian clock. *Proceedings of the National Academy of Sciences of the United States of America*, **100**, 14806-14811.
9. Mirsky, H.P., Liu, A.C., Welsh, D.K., Kay, S.A. and Doyle, F.J., 3rd. (2009) A model of the cell-autonomous mammalian circadian clock. *Proceedings of the National Academy of Sciences of the United States of America*, **106**, 11107-11112.
10. Tigges, M., Marquez-Lago, T.T., Stelling, J. and Fussenegger, M. (2009) A tunable synthetic

mammalian oscillator. *Nature*, **457**, 309-312.

11. Mondragon-Palomino, O., Danino, T., Selimkhanov, J., Tsimring, L. and Hasty, J. (2011) Entrainment of a population of synthetic genetic oscillators. *Science*, **333**, 1315-1319.

12. Hong, C.I., Conrad, E.D. and Tyson, J.J. (2007) A proposal for robust temperature compensation of circadian rhythms. *Proceedings of the National Academy of Sciences of the United States of America*, **104**, 1195-1200.

Supplementary Figures

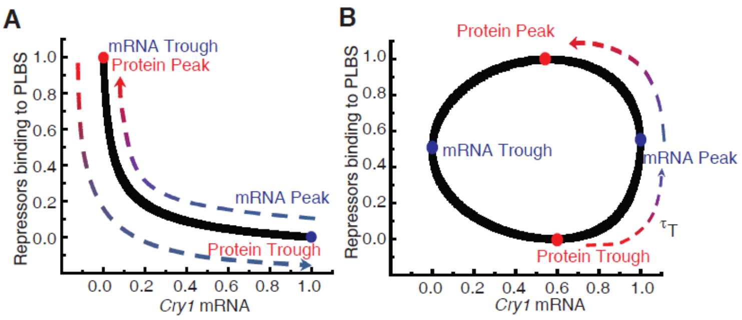

Figure S1 The auxiliary loop coordinates with the primary loop to provide the transcriptional time delay. **(A-B)** The simulation results of the comprehensive model. **(A)** Phase plot between *Cry1* mRNA and repressors binding to PLBS in the system with primary loop only. The repressor binding to PLBS is the comprehensive repression effect. The peak of repressors binding to PLBS coincides with the trough of *Cry1* mRNA, and the trough of repressors binding to PLBS coincides with the peak of *Cry1* mRNA. (The related parameters: $\alpha_1 = 0.09$, $\alpha_2 = 0.018$, $\alpha_3 = 0.0009$, $\alpha_4 = 0.036$, $\beta = 0$). **(B)** Phase plot between *Cry1* mRNA and repressor binding to PLBS after introducing the auxiliary loop. the peak of *Cry1* mRNA is separated from the trough of repressors binding to PLBS (The related parameters: $\alpha_1 = 0.09$, $\alpha_2 = 0.018$, $\alpha_3 = 0.0009$, $\alpha_4 = 0.036$).

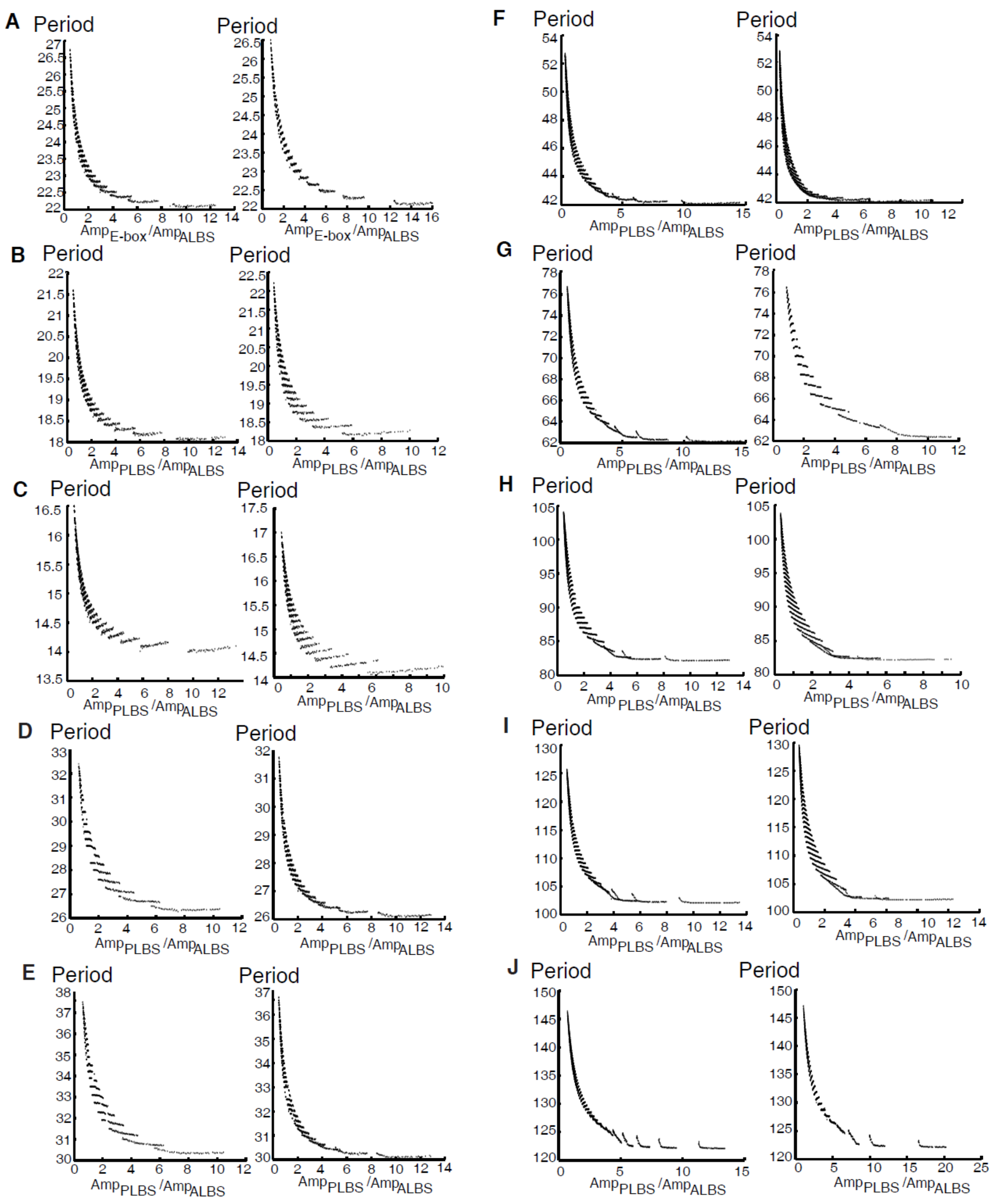

Figure S2 The amplitude ratio is reversely related to the period length in a broader parameter space with fixed τ_P and τ'. Ten parameter sets are randomly selected from the uniform distribution in each pair of time delays and each panel exhibits two typical ones. **(A)**When $\tau_P = 10$ and $\tau' = 4.5$, the period approximately ranges from 23h~29.5h. **(B)** When $\tau_P = 8$ and $\tau' = 3.5$, the period approximately ranges from 18.5h~23.5h. **(C)** When $\tau_P = 6$ and $\tau' = 2.5$, the period approximately ranges

from 14.5h~18h. **(D)** When $\tau_P = 12$ and $\tau' = 5.5$, the period approximately ranges from 26h~35h. **(E)** When $\tau_P = 14$ and $\tau' = 6.5$, the period approximately ranges from 31h~40.5h. **(F)** When $\tau_P = 20$ and $\tau' = 9.5$, the period approximately ranges from 42h~53h. **(G)** When $\tau_P = 30$ and $\tau' = 14.5$, the period approximately ranges from 62h~77h. **(H)** When $\tau_P = 40$ and $\tau' = 19.5$, the period approximately ranges from 82h~105h. **(I)** When $\tau_P = 50$ and $\tau' = 24.5$, the period approximately ranges from 102h~130h. **(J)** When $\tau_P = 60$ and $\tau' = 29.5$, the period approximately ranges from 122h~148h.

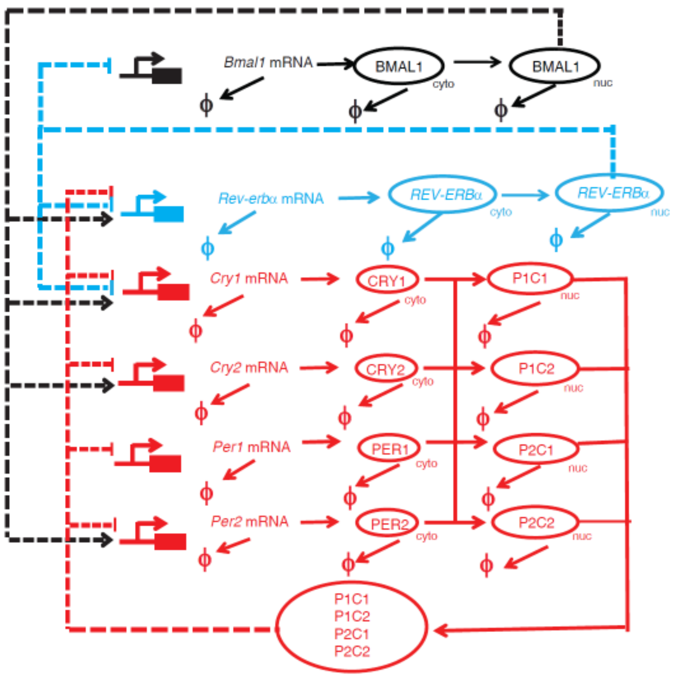

Figure S3 Schematic view of the comprehensive model. *Bmal1* gene translates to BMAL1 protein and goes through a series of processes to activate the transcriptions of *Per1*, *Per2*, *Cry1*, *Cry2* and *Rev-erb*α genes (the black lines). The red lines represent the primary negative feedback loop in circadian clock: PER and CRY proteins form the complexes to inhibit the transcriptions of their own genes. The blue lines represent a positive auxiliary loop: CRY1 inhibits the transcription of *Rev-erb*α

gene, and REV-ERBα feeds back to suppress the expression of *Cry1* gene through RORE.

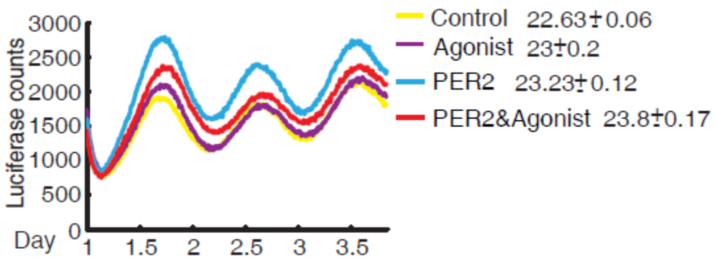

Figure S4 Manipulating the circadian period in PER2-PPARα loop. Bioluminescence results from a representative experiment 48 h after cells were cotransfected with the indicated expression vectors and treated with PPARα agonist (0.8 μm fenofibrate). All experiments were repeated at least three times, and at least three replicates were performed for each group.

Supplementary Tables

Table S1 Reference parameter values for the conceptual model

Parameter	Value	Description
k1	1	The binding coefficient of the repressor to PLBS
k2	0.15	The transfer coefficient from the inactive state to the active state of PLBS
k3	1	The binding coefficient of the repressor to ALBS
k4	0.01	The transfer coefficient from the inactive state to the active state of ALBS
k_P	1	The proportion of the transcription efficiency in *R1* gene enhanced by PLBS to PLBS activity
k_A	0.3	The proportion of the transcription efficiency in *R1* gene enhanced by ALBS to ALBS activity
ks	3	The translation coefficient of *R1* mRNA
kd	1	The degradation coefficient of cytoplasmic R1 protein
τ_P	10	Post-translational time delay
τ'	4.5	The time delay of cytoplasmic R1 protein repressing the nuclear R2 protein
K_P	0.28 ~ 1	The constant which controls the oscillation of the negative primary loop
K_A	0 ~ 6.5	The constant which controls the oscillation of the

		positive auxiliary loop

Table S2 Reference parameter values for the comprehensive model

Parameter	Value	Description	**Period variation (70%)**	**Period variation (130%)**
k1	0.1	The binding coefficient of nuclear BMAL1 to PLBS	0	-0.0416493%
k2	10	The transfer coefficient from the inactive state to the active state of RORE	1.541%	-1.249%
k_PLBSp	1	The proportion of the transcription efficiency enhanced by PLBS in *Per1* to the PLBS activity	1.958%	-0.75%
kp	10000	The transcription coefficient of *Per1* due to PLBS	1.041%	-0.583%
V_Per	7	The translation coefficient of *Per1* mRNA	-0.208%	0.0832986%
kd_Perc	1.8	The degradation coefficient of cytoplasmic PER1	3.082%	-1.666%
k_PLBSp2	1	The proportion of the	0.583%	-0.541%

		transcription efficiency enhanced by PLBS in *Per2* to the PLBS activity		
kp2	10000	The transcription coefficient of *Per2* due to PLBS	0.333%	-0.25%
V_Per2	8	The translation coefficient of *Per2* mRNA	0.125%	-0.0832986%
kd_Per2c	1.8	The degradation coefficient ofcytoplasmic PER2	0.375%	-0.0832986%
k_PLBSc	0.6	The proportion of the transcription efficiency enhanced by PLBS in *Cry1* to the PLBS activity	2.499%	-1.208%
k_ROREc	0.8	The proportion of the transcription efficiency enhanced by RORE in Cry*1* to the RORE activity	-2.416%	3.082%
kc	10000	The transcription coefficient of *Cry1* due to PLBS and RORE	-0.875%	0.541%
V_Cry	7	The translation coefficient	-0.875%	0.541%

		of *Cry1* mRNA		
kd_Cryc	1.8	The degradation coefficient of cytoplasmicCRY1	0.583%	-0.666%
k_PLBSc2	1	The proportion of the transcription efficiency enhanced by PLBS in *Cry2* to the PLBS activity	0.875%	-0.875%
kc2	10000	The transcription coefficient of *Cry2* due to PLBS	0.541%	-0.458%
V_Cry2	5	The translation coefficient of *Cry2* mRNA	0.666%	-0.541%
kd_Cry2c	1.8	The degradation coefficient of cytoplasmicCRY2	-0.583%	0.416%
k_PLBSr	1	The proportion of the transcription efficiency enhanced by PLBS in *Rev-erbα* to the PLBS activity	-2.666%	1.958%
kr	10000	The transcription coefficient of *Rev-erbα* due to PLBS	-1.666%	1.208%

V_Rev	10	The translation coefficient of *Rev-erbα* mRNA	-1.624%	1.208%
kd_Revc	1	The degradation coefficient of cytoplasmic REV-ERBα	0.167%	-0.666%
k_ROREb	1	The proportion of the transcription efficiency enhanced by RORE in *Bmal1* to the RORE activity	-0.167%	0.0832986%
kb	12	The transcription coefficient of *Bmal1* due to RORE	0.0416493%	-0.0416493%
V_Bmal1	0.1	The translation coefficient of *Bmal1* mRNA	0%	-0.0416493%
kd_Bmal1c	0.5	The degradation coefficient of cytoplasmicBMAL1	0.125%	-0.0416493%
ks_P1C1	1.8	The association coefficient for nuclear PER1:CRY1 complex	-0.791%	0.416%
kd_P1C1	1.25	The degradation coefficient for nuclear PER1:CRY1 complex	5.289%	-2.749%

ks_P2C1	1.5	The association coefficient for nuclear PER2:CRY1 complex	-0.0416493%	0.0416493%
kd_P2C1	1.4	The degradation coefficient for nuclear PER2:CRY1 complex	0.167%	-0.0416493%
ks_P1C2	2	The association coefficient for nuclear PER1:CRY2 complex	0.541%	-0.458%
kd_P1C2	2	The degradation coefficient for nuclear PER1:CRY2 complex	-0.25%	0.292%
ks_P2C2	1	The association coefficient for nuclear PER2:CRY2 complex	0.125%	-0.125%
kd_P2C2	2	The degradation coefficient for nuclear PER1:CRY2 complex	0.292%	-0.0416493%
K_{Bmal1}	1	The proportion of nuclear BMAL1 to cytoplasmic BMAL1	0	-0.0416493%
K_{Rev}	1	The proportion of nuclear	1.541%	-1.249%

		REV-ERBα to cytoplasmic REV-ERBα		
K_Bmal1	0.1	Michaelis constant for the *Bmal1* transcription due to pre-mRNA	0.0832986%	-0.0832986%
K_Per	100	Michaelis constant for the *Per1* transcription due to pre-mRNA	-0.708%	1.499%
K_Per2	100	Michaelis constant for the *Per2* transcription due to pre-mRNA	-0.666%	0.416%
K_Cry	100	Michaelis constant for the *Cry1* transcription due to pre-mRNA	1.125%	-1.333%
K_Cry2	100	Michaelis constant for the *Cry2* transcription due to pre-mRNA	-1.208%	0.708%
K_Rev	100	Michaelis constant for the *Rev-erbα* transcription due to pre-mRNA	2.207%	-2.166%
v_{0p}	0.01	The basic transcription rate of *Per1* gene	-0.708%	1%

v_{0c1}	0.01	The basic transcription rate of *Cry1* gene	0	-0.0416493%
v_{0p2}	0.056	The basic transcription rate of *Per2* gene	-0.208%	0.208%
v_{0c2}	0.4	The basic transcription rate of *Cry2* gene	0.125%	-0.167%
v_{0b}	8	The basic transcription rate of *Bmal1* gene	-0.0416493%	-0.0416493%
v_{0r}	0.0001	The basic transcription of rate *Rev-erbα* gene	0	-0.041693%
α_1	0.75	The binding coefficient of nuclear PER1:CRY1 to PLBS	-0.791%	0.458%
α_2	0.15	The binding coefficient of nuclear PER1:CRY2 to PLBS	0.125%	-0.125%
α_3	0.0075	The binding coefficient of nuclear PER2:CRY1 to PLBS	0.541%	-0.458%
α_4	0.3	The binding coefficient of nuclear PER2:CRY2 to PLBS	-0.0416493%	0.0416493%

β	5	The binding coefficient of nuclear REV-ERBα to RORE	-1.624%	1.208%
τ_1	12	The post-translational time delay of cytoplasmic BMAL1	-0.458%	0.541%
τ_2	10	The post-translational time delay of cytoplasmic PER1	-25.489%	18.534%
τ_3	10	The post-translational time delay of cytoplasmic PER2	-3.8%	4.16%
τ_{per2}	0.8	The resultant phase of the unclear regulations in *Per2* mRNA	-0.33%	0.29%
τ_4	9.8	The post-translational time delay of cytoplasmic CRY1	-1.666%	-7.58%
τ_5	9.8	The post-translational time delay of cytoplasmic CRY2	-1.208%	1.791%
τ_6	5	The post-translational time delay of cytoplasmic REV-ERBα	1.46%	-9.78%

τ_{rev}	16	The resultant phase of the unclear regulations in *Per2* mRNA	1.08%	10.37%

Table S3 The comparison of the period length between the experimental data and the simulation results

Genotype	Experiment	Simulation	Relative Parameters
WT	23.7	24.01	Table S2
Pe1$^{-/-}$	22.7	23.1	$\alpha_1 = 0$, $\alpha_2 = 0$
Per2$^{-/-}$	23.5	24.1	$\alpha_3 = 0$, $\alpha_4 = 0$
Cry1$^{-/-}$	22.5	22.2	$\alpha_1 = 0$, $\alpha_3 = 0$
Cry2$^{-/-}$	24.5	24.3	$\alpha_2 = 0$, $\alpha_4 = 0$
Rev-erb$\alpha^{-/-}$	23.4	23.23	V_Rev=2
Fbxl3$^{-/-}$	27.6	27.26	α_1 =5.025, $\alpha_3 = 0.0502$, $\beta = 17.5$
Fbxl3$^{-/-}$; *Rev-erb*$\alpha^{-/-}$	23.7	24	$\alpha_1 = 5.025$, $\alpha_3 = 0.0502$, $\beta = 17.5$, V_Rev=3.7
Bmal1$^{-/-}$	arhythmic	arhythmic	$\frac{d\mathrm{BMAL1_C}}{dt} = 0$, $\mathrm{BMAL1_C}(0) = 0$

Table S4. The comparison of the mRNA phase between the experimental data

and the simulation results in WT case

Gene expression	Simulation result	Experimental data
Per1	CT 12	CT 12h
Per2	CT 12.8	CT 12~16h
Cry1	CT 14.5	CT 16
Cry2	CT 12	CT 12
Bmal1	CT 21.7	CT 20~24
Rev-erbα	CT 4	CT 4~8
